# Reactivity of Ammonia in 1,2-Addition to Group 13 Imine Analogues with G13–P–Ga Linkages: The Electronic Role of Group 13 Elements

**DOI:** 10.3390/molecules30153222

**Published:** 2025-07-31

**Authors:** Zheng-Feng Zhang, Ming-Der Su

**Affiliations:** 1 Department of Applied Chemistry, National Chiayi University, Chiayi 60004, Taiwan; ftg17669@gmail.com; 2 Department of Medicinal and Applied Chemistry, Kaohsiung Medical University, Kaohsiung 80708, Taiwan

**Keywords:** imine analogues, Group 13 elements, ammonia, 1,2-addition reactions

## Abstract

Using density functional theory (M06-2X-D3/def2-TZVP), we investigated the 1,2-addition reactions of NH_3_ with a series of heavy imine analogues, **G13=P-Rea** (where G13 denotes a Group 13 element; Rea = reactant), featuring a mixed G13–P–Ga backbone. Theoretical analyses revealed that the bonding nature of the G13=P moiety in **G13=P-Rea** molecules varies with the identity of the Group 13 center. For G13=B, Al, Ga, and In, the bonding is best described as a donor–acceptor (singlet–singlet) interaction, whereas for G13=Tl, it is characterized by an electron-sharing (triplet–triplet) interaction. According to our theoretical studies, all **G13=P-Rea** species—except the Tl=P analogue—undergo 1,2-addition with NH_3_ under favorable energetic conditions. Energy decomposition analysis combined with natural orbitals for chemical valence (EDA–NOCV), along with frontier molecular orbital (FMO) theory, reveals that the primary bonding interaction in these reactions originates from electron donation by the lone pair on the nitrogen atom of NH_3_ into the vacant p-π* orbital on the G13 center. In contrast, a secondary, weaker interaction involves electron donation from the phosphorus lone pair of the **G13=P-Rea** species into the empty σ* orbital of the N–H bond in NH_3_. The calculated activation barriers are primarily governed by the deformation energy of ammonia. Specifically, as the atomic weight of the G13 element increases, the atomic radius and G13–P bond length also increase, requiring a greater distortion of the H_2_N–H bond to reach the transition state. This leads to a higher geometrical deformation energy of NH_3_, thereby increasing the activation barrier for the 1,2-addition reaction involving these Lewis base-stabilized, heavy imine-like **G13=P-Rea** molecules and ammonia.

## 1. Introduction

In recent decades, the structural and electronic diversity of main-group multiple bonds has undergone a remarkable expansion, driven primarily by the development of sterically encumbering ligands and donor–acceptor stabilization strategies [1,2,3,4]. These advances have enabled the isolation of otherwise highly reactive low-valent species, including a growing family of Lewis base (LB)-stabilized heavy imine analogues featuring Group 13=Group 15 (G13=G15) multiple bonds [5,6,7,8,9,10,11,12,13,14,15,16,17,18,19,20,21,22,23,24,25]. Notably, several synthetic laboratories have successfully prepared and characterized such compounds, offering valuable insights into the bonding capabilities of main-group elements [26,27,28]. These species not only challenge the classical paradigms of multiple bonding but also provide a versatile platform for probing structure–reactivity relationships in main-group chemistry. The stabilization of G13=G15 bonded systems exemplifies the effective use of ligand frameworks to access new bonding motifs, ultimately expanding the frontier of inorganic and organometallic main-group chemistry [29,30,31,32].

The activation of unsaturated molecules through reversible addition to reactive metal centers is a well-established domain in transition metal chemistry and plays a central role in catalytic transformations [33,34,35]. In contrast, comparable reactivity at main-group element centers has remained relatively underexplored, although it has recently attracted growing attention [36,37]. This resurgence of interest stems not only from fundamental curiosity but also from efforts to endow main-group element complexes with transition metal-like reactivity, including catalytic behavior. Of particular interest is the activation of both polar and non-polar E–H bonds (E = H, N, O, etc.) by main-group compounds, a transformation typically facilitated by transition metals. In previous studies [38,39], homonuclear heavier Group 14 (G14) analogues of alkenes (R_2_G14=G14R_2_) have been intensively investigated for such purposes. Their relatively small and tunable HOMO–LUMO energy gaps, coupled with a biradical-type bonding character, make them promising candidates for small-molecule activation [34,40,41,42]. In parallel, heteroatomic double bonds formed between G13 and G15 elements have gained increasing attention due to their valence isoelectronic relationship with G14=G14 alkenes. The inherent polarity of these G13=G15 bonds, arising from the electronegativity difference between the two elements, imparts unique electronic and chemical properties that distinguish them from their carbon-based analogues [5,43,44].

**Scheme 1:**
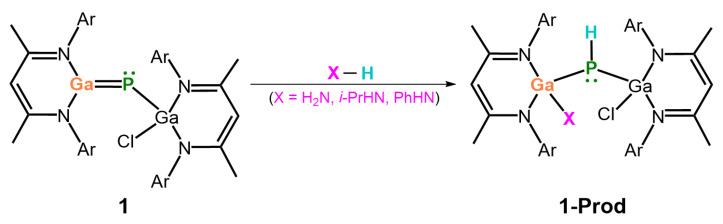
Experimentally observed 1,2-addition reactions of gallaphosphene **1** with NH_3_, *i*-PrNH_2_, and PhNH_2_ under ambient conditions.

**Figure 1:**
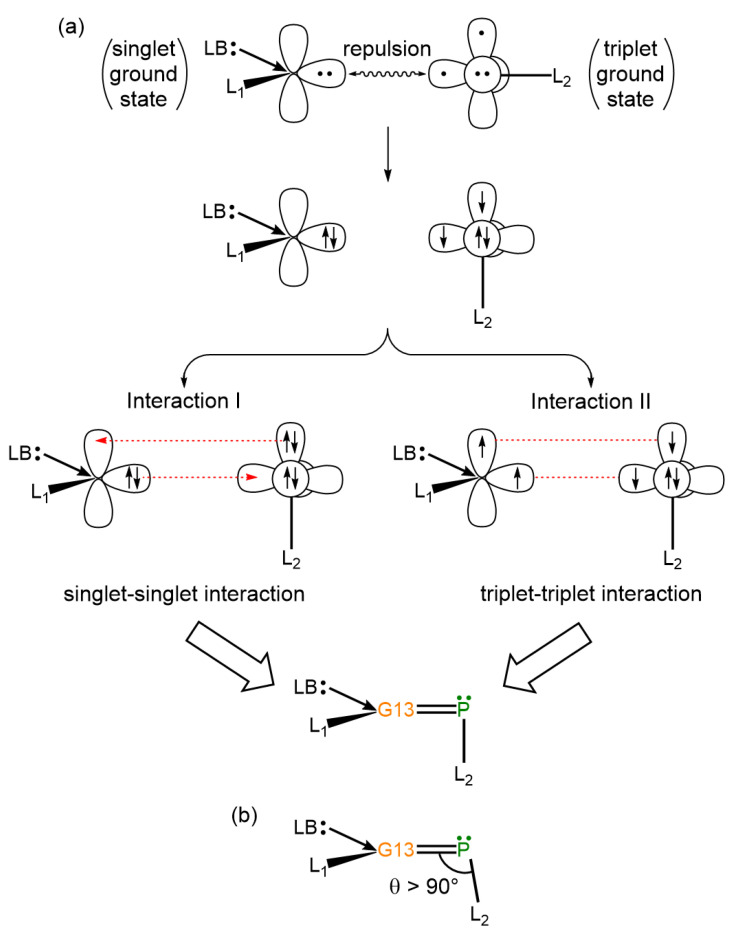
(**a**) Valence bond representations of the doubly bonded **G13=P-Rea** imine-like compound, ((LB:→)(L_1_)G13=P–L_2_ and a comparison between Interaction I (donor–acceptor model) and Interaction II (electron-sharing model). (**b**) VB analysis predicts that the ∠G13–P–L_2_ bond angle in the Lewis base-stabilized, doubly bonded G13=P species is theoretically greater than 90° under realistic molecular conditions.

The selective reactivity of multiply bonded main-group species continues to reveal distinctive bonding patterns and novel activation pathways. A notable example is the recent work by Schulz and co-workers [30,45], who synthesized a gallaphosphene compound (**1**) featuring a Ga–P–Ga chain with a central Ga=P double bond. This species undergoes highly selective 1,2-addition reactions with a range of X–H bonds (X = H_2_N, *i*-PrHN, PhHN) at room temperature (Scheme 1). In each case, the Ga atom acts as the electrophilic site, while the P atom becomes protonated, affording the corresponding 1,2-addition product (**1-Prod**), as illustrated in Figure 1. It is important to highlight that the functionalization of ammonia (NH_3_) represents a particularly challenging and significant transformation in organic chemistry, due to the high bond dissociation energy of the N–H bond [46].

N–H bond activation can occur via either homolytic or heterolytic cleavage pathways. In homolytic cleavage, the N–H bond dissociates such that each atom retains one electron, leading to the formation of radical species [47,48]. In contrast, heterolytic cleavage involves the unequal distribution of bonding electrons, with both electrons being transferred to one atom, generating a cation and an anion [49,50,51]. Previous studies have demonstrated that, in the presence of an appropriate base in solution, heterolytic N–H bond dissociation is significantly more favorable—by approximately 59.2 to 67.8 kcal/mol—when compared with the homolytic pathway [52]. Traditionally, such activations have been the domain of transition metal complexes; however, recent advances have shown that main-group element complexes can also engage in this reactivity, offering alternative approaches for small molecule activation [33,34,35,36,37,38].

**Scheme 2:**
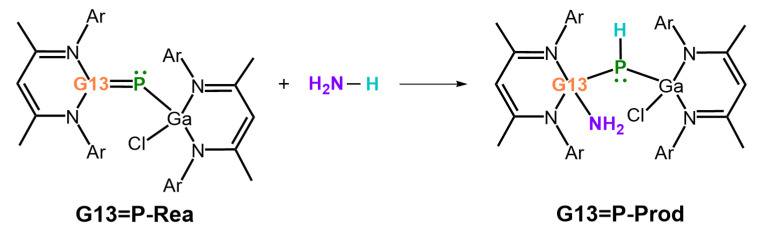
Theoretical model describes a 1,2-addition of ammonia to a Lewis base-stabilized heavy imine analogue (G13=P-Rea).

The above reactivity pattern reflects not only the pronounced polarization of the Ga=P bond but also highlights the potential of heavier main-group elements to participate in ambiphilic interactions and small-molecule activation under mild conditions. These captivating experimental findings [30,45] have sparked our curiosity and inspired further theoretical investigation. Given the practical limitations of experimental reagents and instrumentation, theoretical calculations offer an indispensable platform for rapidly and reliably elucidating reaction mechanisms and predicting molecular behavior. Nevertheless, to the best of our knowledge, no theoretical studies have yet been reported that address the specific experimental observations outlined above [30,45]. Motivated by these results, we adopted the gallaphosphene-based system as a computational model to examine how the identity of the Group 13 element influences the reactivity of Lewis base (LB)-stabilized heavy imine-like compounds in their 1,2-addition reactions with ammonia [53,54,55]. Our investigation focused on elucidating the bonding characteristics and reactivity of LB-supported imine-like species containing a G13–P–Ga framework, and on comparing their reactivity with that of analogous imine species. To this end, we employed density functional theory (DFT), in conjunction with advanced computational techniques, to study the formation of the 1,2-addition product between LB-stabilized heavy imine-like molecules (**G13=P-Rea**; Rea = reactant) and NH_3_, as illustrated in Scheme 2. The goal of this study is to identify the key factors that govern both the reactivity and activation energy of these transformations. Ultimately, we hope that our theoretical insights will serve as a valuable foundation for the development of more efficient synthetic methodologies and the exploration of broader applications in main-group chemistry.

## 2. Results and Discussion

*(1) The Nature of the G13=P Bonding in G13=P-Rea*

We begin by examining the intrinsic bonding characteristics of the G13=P double bond in the **G13=P-Rea** species, prior to conducting our theoretical investigation into the 1,2-addition reaction of the H_2_N–H bond with doubly bonded G13=P imine-like compounds (Scheme 1). The general structure of the **G13=P-Rea** molecule can be represented as (LB:→)(L_1_)G13=P–L_2_, in which LB acts as a strong two-electron donor, while the conventional ligands L_1_ and L_2_ each contribute one electron to the overall bonding framework.

It is worth noting that the conclusion derived from valence bond (VB) theory, as illustrated in Figure 1b, suggests that the ∠G13–P–L_2_ bond angle in the LB-stabilized, doubly bonded imine-like G13=P species—formally denoted as (LB:→)(L_1_)G13=P–L_2_—is predicted to exceed 90°. This is attributed to steric repulsions between the lone pair electrons on the phosphorus center and the bulky substituents under realistic conditions. This theoretical prediction is strongly supported by available X-ray crystallographic data [30,32,45], which closely align with the geometry proposed by our VB-based structural analysis.

To describe the bonding nature of the central G13=P double bond, the LB-supported imine-like molecule ((LB:→)(L_1_)G13=P–L_2_) can be conceptually separated into two parts: (LB:→)(L_1_)G13: and :P–L_2_. Two principal bonding interactions can be considered. The first, referred to as Interaction I—the donor–acceptor interaction (or singlet–singlet interaction)—involves the interaction of two singlet fragments: [(LB:→)(L_1_)G13:]^1^ + [:P–L_2_]^1^ → [(LB:→)(L_1_)G13=P–L_2_]^1^, as shown schematically in Figure 1a. The second, Interaction II, termed the electron-sharing interaction (or triplet–triplet interaction), involves the coupling of two triplet-state fragments: [(LB:→)(L_1_)G13:]^3^ + [:P–L_2_]^3^ → [(LB:→)(L_1_)G13=P–L_2_]^1^, as represented graphically in Figure 1a. It is important to note that two individual triplet fragments can couple to afford an overall singlet ground state.

**Scheme 3:**
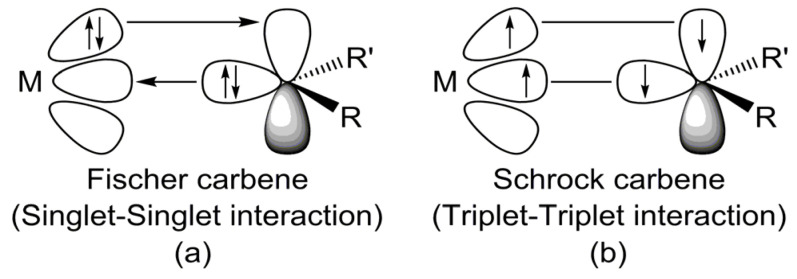
(**a**) The Fischer type and (**b**) the Schrock type metal carbenes.

In fact, the above two interaction models closely resemble the bonding paradigms of Fischer-type [56] and Schrock-type [57] metal carbenes, respectively (Scheme 3), which are well-known and extensively studied in organometallic chemistry.

A detailed bonding analysis of the G13=P linkage in the **G13=P-Rea** molecules was performed using the EDA method. As emphasized in prior theoretical studies [58], the orbital interaction energy (Δ*E*_Orb_) serves as a critical indicator for evaluating the intrinsic nature of the bonding interaction, reflecting the stabilization gained from orbital mixing between fragments. A lower absolute |Δ*E*_Orb_| value typically indicates a more accurate electronic description of the interacting fragments [58]. As shown in Table 1, our M06-2X-D3 calculations reveal that, with the exception of **Tl=P-Rea**, the absolute |Δ*E*_Orb_| values for the triplet–triplet (electron-sharing) interactions are significantly lower (145.8–210.3 kcal/mol) than those for the singlet–singlet (donor–acceptor) interactions (213.9–286.1 kcal/mol). This finding indicates that the bonding character of the G13=P double bond in LB-stabilized, imine-like **G13=P-Rea** species (G13=B, Al, Ga, and In) is primarily dominated by electron-sharing interactions.

In contrast, EDA analysis for **Tl=P-Rea** reveals that the absolute |Δ*E*_Orb_| values of the singlet–singlet (178.9 kcal/mol) and triplet–triplet (179.9 kcal/mol) interactions are nearly identical. This observation implies that the bonding nature of the Tl=P double bond in **Tl=P-Rea** can be described either by a donor–acceptor formulation, (LB:→)(L_1_)Tl⇌P–L_2_, or by a classical double bond structure, (LB:→)(L_1_)Tl=P–L_2_, where (LB:→)(L_1_) = CH[C(Me)NAr]_2_ (Ar = 2,6-*i*Pr_2_C_6_H_3_) and L_2_ = Ga(Cl)(LB:→)(L_1_), as illustrated in Scheme 1.

In classical main-group chemistry, multiple bonding involving Group 13 elements is relatively uncommon due to the limited availability of low-lying p orbitals and poor π-overlap capability. However, recent investigations into LB-stabilized G13=P systems have uncovered notable deviations from traditional double-bond models [26,27,28,29,30,31,32]. To further probe the intrinsic electronic properties of the G13–P linkage in these heavy imine-like **G13=P-Rea** molecules, a detailed natural bond orbital (NBO) [59] analysis was carried out.

The results, presented in Table 2, demonstrate that the G13=P bonds are highly polarized and electronically weak. Specifically, the σ-bonds are polarized toward the phosphorus atom, with polarization values ranging from 41.32% to 69.43%, while the π-bonds show even higher degrees of polarization, ranging from 74.87% to 85.97%. The computed G13–P (G13=B, Al, Ga, and In) bond orders lie between 1.371 and 1.619—significantly lower than the ideal bond order of 2.0 expected for a classical double bond. These findings suggest that the central G13=P linkage in the doubly bonded imine-like **G13=P-Rea** (G13=B, Al, Ga, and In) molecules—characterized by a mixed G13–P–Ga framework—possesses only a partial double-bond character. It should be noted that the NBO calculation results (Table 2) provide only the σ-bond-related data for the **Tl=P-Rea** molecule, without any indication of a corresponding π-bond contribution. Moreover, the calculated bond order for the central Tl–P bond is 1.262. These theoretical findings strongly suggest that the Tl and P centers in this LB-stabilized, imine-like **Tl=P-Rea** molecule are connected only via a Tl–P single bond. Overall, the NBO analysis highlights that the G13=P bonds in these LB-supported, heavier imine-type systems are relatively weak and electronically fragile, implying their potential susceptibility to bond cleavage under specific chemical conditions.

*(2) 1,2-Addition Reactions of G13=P-Rea with Ammonia*

**Figure 2:**
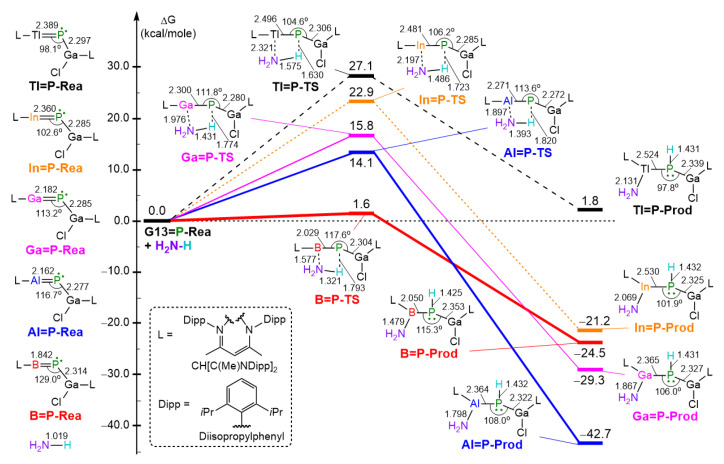
Computed free energy surface for the 1,2-addition of NH_3_ to **G13=P-Rea** ((L)G13=P–Ga(Cl)(L); L = CH[C(Me)NAr]_2_, Ar = 2,6-*i*Pr_2_C_6_H_3_) at the M06-2X-D3/def2-TZVP level of theory. Bond lengths are given in Å, and relative free energies are reported in kcal/mol.

Figure 2 displays the optimized geometries of the reactants (**G13=P-Rea** + NH_3_), transition states (**G13=P-TS**), and products (**G13=P-Prod**) involved in the 1,2-addition reactions of LB-supported, doubly bonded heavy imine-like molecules (**G13=P-Rea**) with NH_3_, calculated at the **M06-2X-D3/def2-TZVP** level of theory.

The optimized geometries of the **G13=P-Rea** species shown in Figure 2 exhibit notable deviations from the idealized bond angles predicted by VB theory (Figure 1a), which suggests a perfectly perpendicular arrangement (∠G13–P–L_2_ = 90°). However, due to steric repulsion between the lone pair on the P atom and the bulky substituents present under realistic molecular conditions, the ∠G13–P–L_2_ bond angle is expected to be greater than 90° (Figure 1b). Indeed, our computational results, shown in Figure 2, confirm that all ∠G13–P–Ga bond angles in the current **G13=P-Rea** systems, which feature a mixed G13–P–Ga framework, are significantly larger than 90°. Such angular distortions are characteristic of systems involving mixed-main-group centers and demonstrate how ligand-induced steric constraints can modulate molecular geometry and potentially influence reaction pathways. In addition, the computed ∠G13–P–Ga bond angles exhibit a clear decreasing trend along the Group 13 series: **B=P-Rea** (129.0°) **> Al=P-Rea** (116.7°) **> Ga=P-Rea** (113.2°) **> In=P-Rea** (102.6°) **> Tl=P-Rea** (98.1°). In other words, as the atomic weight of the G13 element increases, the corresponding ∠G13–P–Ga bond angle in the **G13=P-Rea** molecule becomes smaller. This trend can be attributed to the “relativistic inert s-pair effect” or the so-called “orbital non-hybridization effect” [60,61,62,63]. Specifically, as the G13 element becomes heavier, the inner s orbital contracts more due to relativistic effects, while the outer p orbital remains relatively unaffected. This results in a poor energetic and spatial match between s and p orbitals, reducing their hybridization efficiency. Consequently, heavier G13 elements tend to form more acute ∠G13=P–Ga bond angles due to the dominance of non-hybridized orbital interactions [60,61,62,63].

Despite thorough exploration, no stable van der Waals-type precursor complex was located prior to the transition state (**G13=P-TS**), suggesting that the reaction proceeds through a concerted mechanism. As shown in Figure 2, the 1,2-addition reaction follows a pathway involving a pseudo-four-membered-ring transition state (**G13=P-TS**), ultimately leading to product formation (**G13=P-Prod**) in which both the G13–N and P–H bonds are formed in a synchronous fashion. As shown in Figure 2, our DFT-calculated results exhibit a clear increasing trend in the activation free energy (∆*G*_ACT_, kcal/mol) for the 1,2-addition reactions of **G13=P-Rea** with NH_3_: **B=P-TS** (1.6) < **Al=P-TS** (14.1) < **Ga=P-TS** (15.8) < **In=P-TS** (22.9) < **Tl=P-TS** (27.1). This trend indicates that the activation barrier height increases with the atomic number of the G13 element. Furthermore, the corresponding reaction free energies (∆*G*_RXN_) are exergonic (ranging from –42.7 to –21.2 kcal/mol) for all systems except **Tl=P-Rea**, which is endergonic (∆*G*_RXN_ = +1.8 kcal/mol). These theoretical findings suggest that, from both kinetic and thermodynamic perspectives, the 1,2-addition of NH_3_ to the lighter **G13=P-Rea** species (**B=P-Rea**, **Al=P-Rea**, **Ga=P-Rea**, and **In=P-Rea**) is energetically favorable. Notably, our computational predictions are in good agreement with available experimental data [30,45], as illustrated in Scheme 1.

*(3) FMO Analysis*

**Figure 3:**
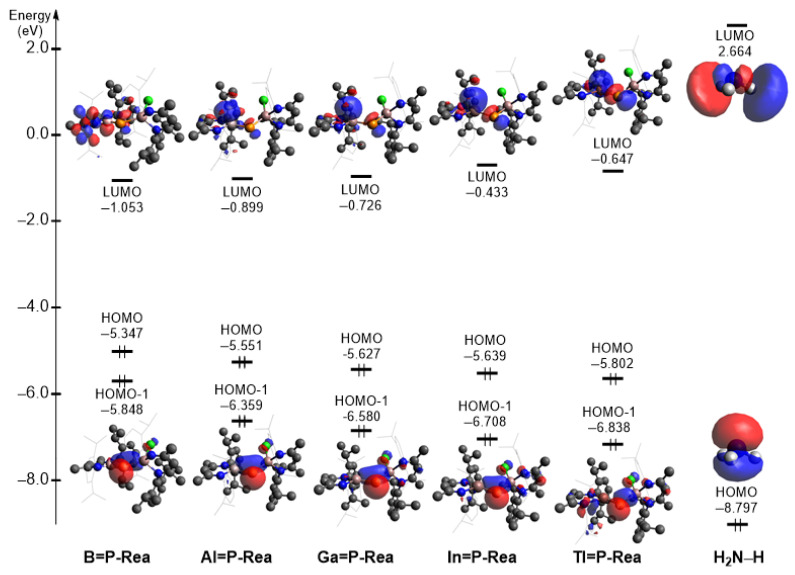
Selected important frontier molecular orbitals and their corresponding energies (in eV) for the LB-stabilized imine-like **G13=P-Rea** ((L)G13=P–Ga(Cl)(L); L = CH[C(Me)NAr]_2_, Ar = 2,6-*i*Pr*_2_*C*_6_*H*_3_*) molecules, featuring a mixed G13–P–Ga backbone, and for ammonia.

To achieve a more in-depth understanding of the bonding characteristics between LB-stabilized imine-like **G13=P-Rea** molecules and NH_3_ in the 1,2-addition reactions, we performed a detailed investigation of their electronic structures based on frontier molecular orbital (FMO) theory [64]. As depicted in Figure 3, the molecular orbital diagrams illustrate the spatial distributions and energy levels (in eV) of the highest occupied molecular orbital (HOMO) and the lowest unoccupied molecular orbital (LUMO) for the **G13=P-Rea** molecules as well as NH_3_. The HOMO-1 of **G13=P-Rea** is primarily a lone pair orbital localized on the Lewis basic phosphorus atom. In contrast, its LUMO corresponds to an unoccupied p-* antibonding orbital delocalized between the G13 and P atoms, with a more substantial orbital contribution from the G13 element. Based on the orbital energy data summarized in Figure 3, Table 3 provides the computed energy differences between the LUMO of NH_3_ and the HOMO-1 of **G13=P-Rea**, as well as between the LUMO of **G13=P-Rea** and the HOMO of NH_3_. For all systems examined, the energy gaps associated with the former interaction (8.512–9.502 kcal/mol) are considerably larger than those of the latter (7.744–8.364 kcal/mol). According to FMO theory, a smaller HOMO–LUMO energy gap generally corresponds to a stronger orbital interaction, whereas a larger gap implies weaker electronic overlap [64,65]. Therefore, the FMO results presented in Table 3 strongly suggest that the dominant orbital interaction in the 1,2-addition reaction occurs between the LUMO of the heavy imine-like **G13=P-Rea** species and the HOMO of NH_3_.

To move beyond qualitative orbital alignment, modern computational approaches such as EDA–NOCV have proven invaluable in providing energy-resolved insights into bonding mechanisms. These methods complement frontier orbital theory by quantifying donor–acceptor interactions in terms of orbital overlap and charge flow.

*(4) EDA–NOCV Analysis*

**Figure 4:**
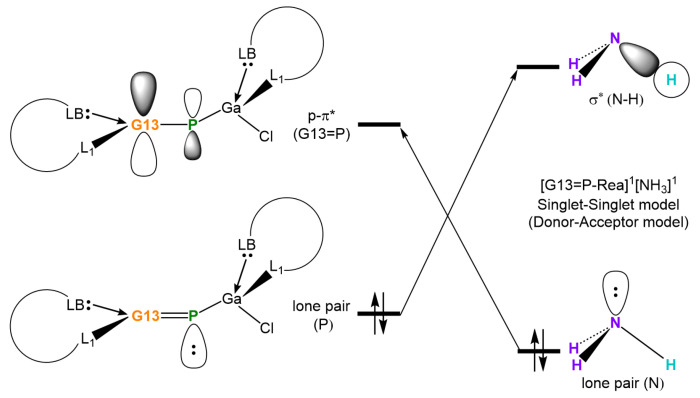
The singlet–singlet interaction or the donor–acceptor interaction for the 1,2-addition reaction between the imine-like **G13=P-Rea** molecule and NH_3_. The **G13=P-Rea** species is represented as (LB)(L_1_)G13=P–Ga(Cl)(LB)(L_1_), where G13 denotes a Group 13 element and (LB)(L_1_) refers to the CH[C(Me)NAr]_2_ (Ar = 2,6-*i*Pr_2_C_6_H_3_) ligand framework.

Before applying the EDA–NOCV approach to investigate the electronic structures of the transition states involved in the 1,2-addition reactions studied herein, we first introduce two conceptual bonding models to determine the most appropriate electronic description of the interaction between the LB-stabilized heavy imine-like **G13=P-Rea** molecules—characterized by a G13–P–Ga backbone—and ammonia. These two bonding models are schematically presented in Figure 4.

The first model is the donor–acceptor interaction (also referred to as the singlet–singlet interaction), where the HOMO-1 of **G13=P-Rea**—primarily consisting of a lone pair on the phosphorus center—interacts with the LUMO of NH_3_, corresponding to the σ* antibonding orbital of the N–H bond. Concurrently, the HOMO of NH_3_, comprising a lone pair on nitrogen, donates electron density into the LUMO of **G13=P-Rea**, which features a vacant p–π* orbital on the G13=P unit, with a more significant orbital contribution from the G13 atom than from phosphorus. This bonding scenario is symbolically denoted as [**G13=P-Rea**]^1^ + [NH_3_]^1^ → [**G13=P-TS**]^1^, as shown in Figure 4.

**Figure 5:**
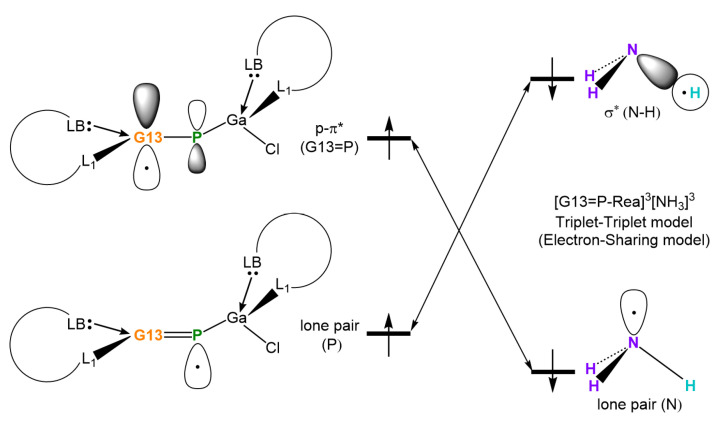
The triplet–triplet interaction or the electron-sharing interaction for the 1,2-addition reaction between the imine-like **G13=P-Rea** molecule and NH_3_. The **G13=P-Rea** species is represented as (LB)(L_1_)G13=P–Ga(Cl)(LB)(L_1_), where G13 denotes a Group 13 element and (LB)(L_1_) refers to the CH[C(Me)NAr]_2_ (Ar = 2,6-*i*Pr_2_C_6_H_3_) ligand framework.

The second model is the electron-sharing interaction (or triplet–triplet interaction), in which both reactants are considered in their triplet states. This leads to a different transition state pathway, represented as [**G13=P-Rea**]^3^ + [NH_3_]^3^ → [**G13=P-TS**]^1^, as illustrated in Figure 5.

In light of the established bonding models for Fischer [56] and Schrock [57] carbenes, which serve as archetypes in transition metal chemistry, we recognize a compelling analogy in the interaction between NH_3_ and LB-stabilized imine-like **G13=P-Rea** systems. This conceptual parallel between main-group and transition metal chemistry provides a valuable framework for interpreting the EDA–NOCV results and deepening our understanding of the electronic contributions to the 1,2-addition process. To assess which bonding description is more appropriate for characterizing the transition states, we carried out EDA–NOCV calculations using **G13=P-Rea** and NH_3_ as interacting fragments in both singlet and triplet spin states (Figure 4 and Figure 5, respectively). The results are summarized in Table 4. According to prior theoretical studies [58], the magnitude of the orbital interaction energy (Δ*E*_Orb_) serves as a key criterion in distinguishing between donor–acceptor and electron-sharing bonding schemes. Our EDA–NOCV results (Table 4) show that the absolute values of Δ*E*_Orb_ for the singlet–singlet interaction (128.8–210.8 kcal/mol) are significantly smaller than those for the triplet–triplet interaction (187.7–355.7 kcal/mol). These findings strongly support the conclusion that the electronic structure of the **G13=P-TS** transition states is more accurately described by the donor–acceptor interaction (singlet–singlet interaction) model rather than the electron-sharing (triplet–triplet interaction) model.

**Figure 6:**
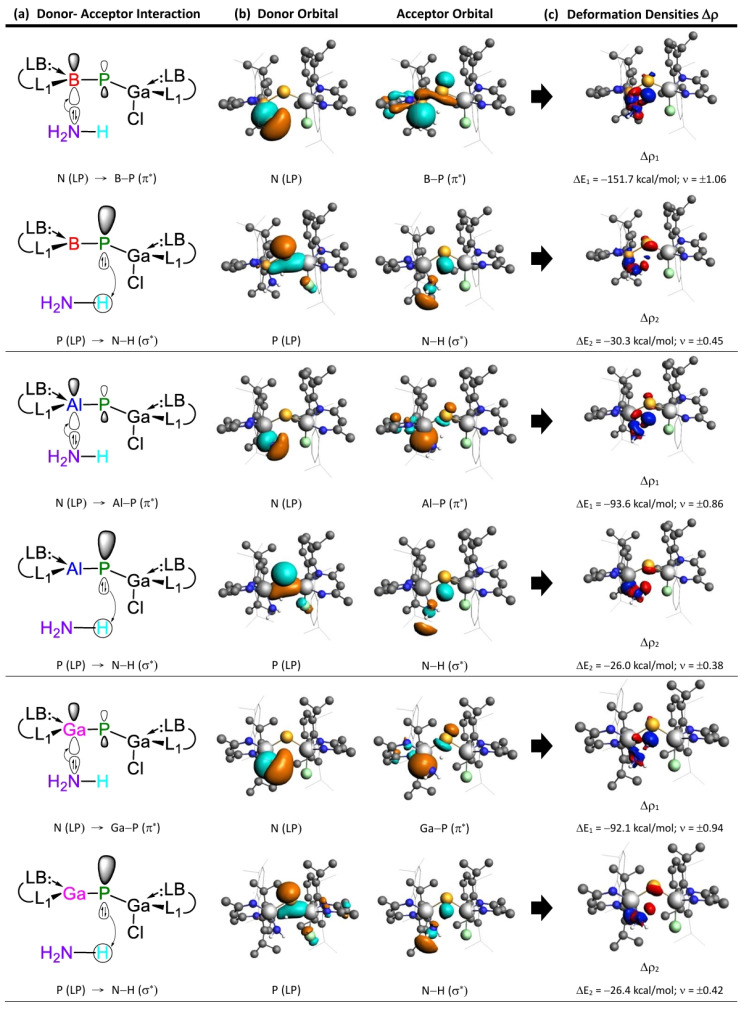

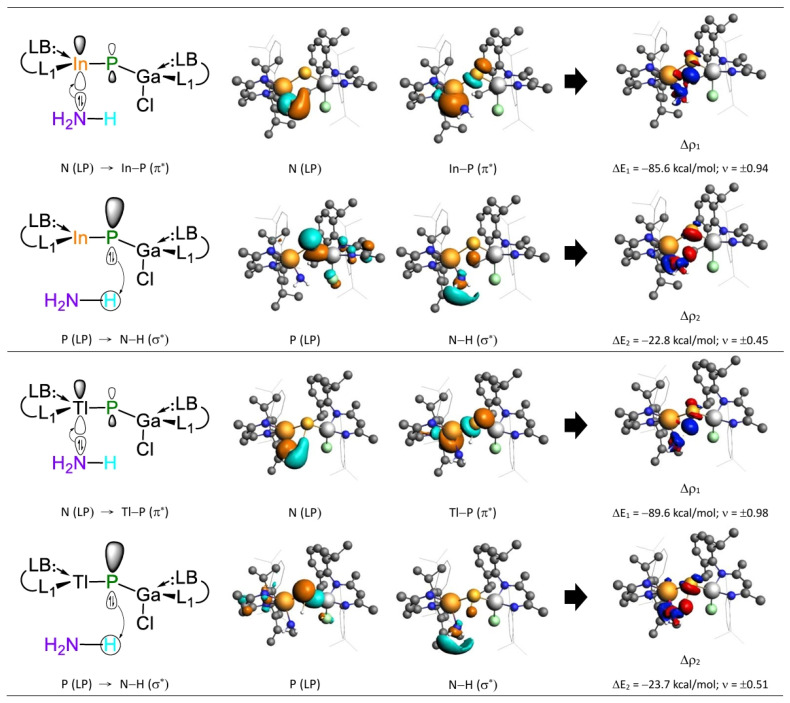
EDA–NOCV analysis of **G13=P-TS**, consisting of (**a**) a qualitative depiction of orbital interactions between **G13=P-Rea** and NH**_3_**; (**b**) the shape of the dominant interaction involving the occupied and vacant orbitals of **G13=P-Rea** and NH**_3_**; and (**c**) plots of deformation densities Δρ_(1)_ and Δρ_(2)_, corresponding to the pairwise orbital interactions between the two closed-shell fragments, along with their respective interaction energies, Δ*E*_Orb(1)_ and Δ*E*_Orb(2)_ (in kcal/mol). The deformation density plots indicate the direction of charge flow, with electron density moving from red (donor) to blue (acceptor) regions.

Traditional bonding analyses often fail to capture the subtle interplay of orbital interactions during bond formation. The EDA–NOCV approach, by resolving total orbital interaction energy into distinct components and visualizing their associated deformation densities, offers a more complete picture of bonding dynamics—particularly relevant for understanding the complex reactivity of main-group transition states. Table 4 shows that the total orbital interaction energy (Δ*E*_Orb_) between singlet fragments in **G13=P-TS** is primarily composed of two major components: Δ*E*_Orb(1)_ and Δ*E*_Orb(2)_. The first component, |Δ*E*_Orb(1)_|, ranges from 85.6 to 151.7 kcal/mol and accounts for 66.3–71.9% of the total |Δ*E*_Orb_|. In contrast, the second component, |Δ*E*_Orb(2)_|, contributes only 22.8 to 30.3 kcal/mol, corresponding to approximately 14.4–19.0% of the total orbital interaction energy. Notably, the EDA–NOCV method provides not only quantitative insights into orbital interactions but also qualitative visualization of electronic structural changes through deformation density maps. These deformation densities offer a detailed view of how electronic distributions are reorganized upon bond formation. The corresponding deformation density plots, Δρ_(1)_ and Δρ_(2)_, are depicted in Figure 6 to illustrate the nature of the dominant orbital interactions.

In the context of main-group chemistry, understanding the nature of donor–acceptor interactions is crucial for rationalizing reactivity trends. As shown in Figure 6, the present NOCV analysis provides compelling evidence that the nitrogen center in NH**_3_** acts as a strong nucleophilic donor, with the LP(N) of NH**_3_** → p–π* (G13) of **G13=P-Rea** interaction serving as the primary driving force for adduct formation. This observation aligns well with the FMO-based predictions, where HOMO–LUMO interactions favor such charge transfer (Table 3). Additionally, although weaker, the back-donation from the phosphorus lone pair of **G13=P-Rea** into the vacant σ* N–H orbital of NH**_3_** contributes to product stabilization. This secondary interaction can be described as the σ* (N–H) of NH**_3_** ← LP(P) of **G13=P-Rea**. Taken together, the FMO (Table 3) and NOCV (Figure 6) analyses provide complementary insights into the orbital origins of reactivity and selectivity in G13=P-based heavy imine-like systems with small-molecule substrates like NH**_3_**.

*(5)* *ASM Analysis*

**Figure 7:**
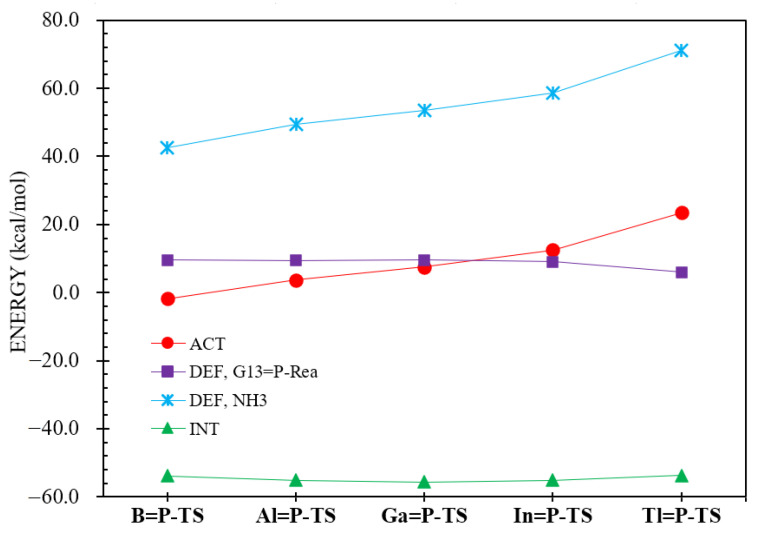
Activation strain analysis plot of Δ*E*_ACT_ (kcal mol^−1^) for the 1,2-addition reaction of NH**_3_** to **G13=P-Rea**. Data extracted from Table 4.

In order to elucidate the origin of the activation barrier associated with the 1,2-addition reaction of NH**_3_** to **G13=P-Rea**, we performed an ASM analysis on the corresponding transition state (**G13=P-TS**), using **G13=P-Rea** and NH**_3_** as the interacting fragments. As depicted in Table 5 and Figure 7, the overall activation energy (∆*E*_ACT_) is predominantly governed by the deformation energy of NH**_3_** (∆*E*_DEF,NH3_), while the deformation energy of **G13=P-Rea** (∆*E*_DEF,G13=P-Rea_) and the interaction energy (Δ*E*_INT_) at the transition state contribute to a lesser extent. This suggests that a significant energetic penalty is associated with distorting NH**_3_** into a geometry suitable for reaction, reflecting the resistance of the nucleophile to undergo structural reorganization. Conversely, the **G13=P-Rea** fragment appears relatively pre-organized, undergoing minimal geometric changes upon activation. The relatively flat profile of Δ*E*_INT_ further suggests that stabilizing orbital interactions are not fully developed at the transition state, thus contributing minimally to the compensation for the associated deformation energy. These observations underscore the central role of NH**_3_** deformation in shaping the reaction barrier and complement the electronic descriptions provided by FMO and NOCV analyses.

The rationale behind this explanation can be understood as follows. As **G13=P-Rea** and NH_3_ approach each other from an infinite separation to undergo the 1,2-addition reaction, both molecules must simultaneously undergo geometric deformations to allow their frontier orbitals to overlap effectively, thereby achieving optimal bonding interactions. Given that **G13=P-Rea** is significantly larger in size compared with NH**_3_**, the smaller NH**_3_** molecule is more susceptible to structural distortion. Consequently, NH**_3_** incurs a greater deformation energy, whereas the deformation energy associated with **G13=P-Rea** remains relatively small. As shown in Figure 7, the activation energy for this 1,2-addition reaction is predominantly governed by the deformation energy of NH**_3_**. Moreover, as the atomic number of the Group 13 element increases, the corresponding atomic radius becomes larger [66], leading to an elongated G13–P bond in the **G13=P-Rea** moiety. To maintain efficient orbital overlap—specifically between the G13 and N atoms and between P and H atoms—the H**_2_**N–H bond in NH**_3_** must also be elongated. This structural adjustment further increases the deformation energy of NH**_3_** (∆*E*_DEF,NH3_), which can serve as a determining factor in modulating the activation barrier of the 1,2-addition reaction.

Further supporting evidence comes from our M06-2X calculations, which reveal that the H**_2_**N–H bond length in the transition structures increases progressively in the order of 21.6% (**B=P-TS**) < 27.7% (**Al=P-TS**) < 31.2% (**Ga=P-TS**) < 36.2% (**In=P-TS**) < 44.3% (**Tl=P-TS**), relative to the bond length in isolated NH_3_ (1.019 Å). This trend strongly suggests that the total deformation energy of NH**_3_** (∆*E*_DEF,NH3_) increases with the atomic number of the Group 13 element, as illustrated in Figure 7. In addition, the theoretical data indicate that the B=P-based heavy imine-like compound (**B=P-Rea**) reaches its corresponding transition state relatively early, while the **Tl=P-Rea** compound—the heaviest in the series—attains its transition state relatively late. According to Hammond’s postulate [67], this implies that the LB-stabilized **B=P-Rea** system undergoes a 1,2-addition reaction with NH**_3_** via a low-energy, exergonic pathway. In contrast, the LB-stabilized **Tl=P-Rea** compound is predicted to exhibit a higher activation barrier and an endergonic N–H bond cleavage process. These theoretical predictions are in excellent agreement with the calculated energetic profiles presented in Figure 2.

## 3. Methodology

All geometry optimizations of stationary points were performed without symmetry constraints at the M06-2X [68] -D3/def2-TZVP [69] level of theory, incorporating Grimme’s D3 dispersion correction [70,71,72] (hereafter denoted as M06-2X-D3/def2-TZVP), as implemented in the Gaussian 16 program package [73]. Based on previous benchmarks [74,75], the M06-2X functional is well suited for describing thermochemistry and reaction kinetics involving main-group elements. The identity of each stationary point on the potential energy surface was verified via analytical frequency calculations at the same level of theory: true minima exhibited no imaginary frequencies, while transition states displayed a single imaginary frequency [76]. Thermodynamic corrections and Kohn–Sham orbital information were extracted from these vibrational analyses. All thermochemical data were reported under standard-state conditions (298.15 K, 1 atm). To obtain more accurate electronic energies, single-point energy calculations were performed using the Amsterdam Density Functional (ADF) [77] program at the ZORA [78,79,80] -M06-2X-D3/TZ2P [81] level of theory, based on the M06-2X-D3/def2-TZVP-optimized geometries. This two-level computational scheme is abbreviated as ZORA-M06-2X-D3/TZ2P//M06-2X-D3/def2-TZVP.

To investigate the origin of the activation barriers in the reactions between G13=P doubly bonded heavy imine-like molecules (**G13=P-Rea**) and NH_3_ at the transition states, the activation strain model (ASM) [82,83,84] was applied to dissect the overall electronic activation energy (Δ*E*_ACT_) into two major components: the strain energy (∆*E*_DEF_ = ∆*E*_DEF,G13=P-Rea_ + ∆*E*_DEF,NH3_) and the interaction energy (Δ*E*_INT_), as evaluated at the ZORA-M06-2X-D3/TZ2P//M06-2X-D3/def2-TZVP level of theory (Equation (1)):

Δ*E*_ACT_ = ∆*E*_DEF_ + Δ*E*_INT_ = ∆*E*_DEF,G13=P-Rea_ + ∆*E*_DEF,NH3_ + Δ*E*_INT_ (1)

Furthermore, the interaction energy Δ*E*_INT_ between the reactant fragments was decomposed into four physically meaningful terms: electrostatic interactions (Δ*E*_Elstat_), Pauli repulsion (Δ*E*_Pauli_), orbital interactions (Δ*E*_Orb_), and dispersion interactions (Δ*E*_Disper_), as shown in Equation (2):

Δ*E*_INT_ = Δ*E*_Elstat_ + Δ*E*_Pauli_ + Δ*E*_Orb_ + Δ*E*_Disper_ (2)

Energy decomposition analysis (EDA) [85,86,87] and natural orbitals for chemical valence (NOCV) [88,89,90,91] analysis were performed via single-point calculations using the ADF program at the ZORA-M06-2X-D3/TZ2P level of theory. Within the NOCV framework, the transition-state structure was fragmented into **G13=P-Rea** and NH_3_ components, allowing for a detailed examination of the bonding and electronic interactions that emerge upon recombination. Charge-transfer pathways and electron redistribution—namely accumulation and depletion of electron density—were visualized through deformation density analysis. For further methodological details and background on these approaches, the reader is referred to relevant literature sources [92,93,94].

## 4. Conclusions

**Figure 8:**
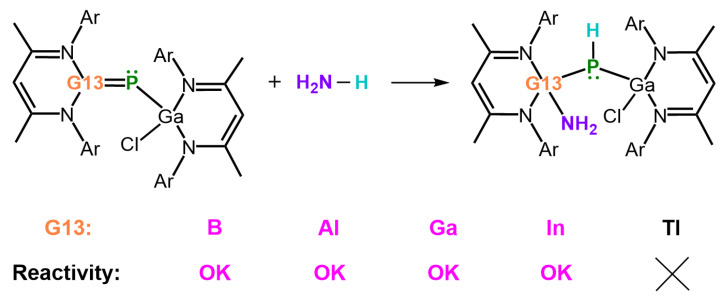
Theoretical predictions of the 1,2-addition reaction between **G13=P-Rea** and NH_3_.

**Figure 9:**
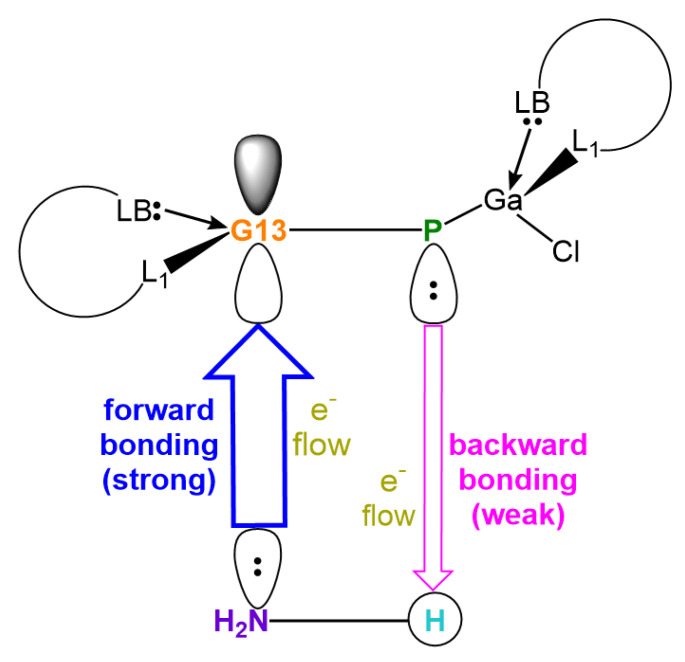
Donor–acceptor bonding interactions between the LB-supported heavy imine-like molecule **G13=P-Rea** and NH_3_.

In this study, a comprehensive theoretical investigation was carried out using a combination of valence bond (VB) theory, natural bond orbital (NBO) analysis, frontier molecular orbital (FMO) theory, energy decomposition analysis with natural orbitals for chemical valence (EDA–NOCV), and the activation strain model (ASM). This analysis aimed to elucidate the mechanistic features of the 1,2-addition reaction between a Lewis base-stabilized, imine-like **G13=P-Rea** species—characterized by G13–P–Ga linkages—and ammonia. Several significant conclusions have been drawn from these findings, as follows:

1. Theoretical insights reveal that electron-sharing (triplet–triplet) interactions primarily dictate the bonding nature of G13=P double bonds in LB-stabilized, imine-like **G13=P-Rea** compounds (G13=B, Al, Ga, and In) and are best described by electron-sharing interactions. However, the Tl=P bond in the **Tl=P-Rea** molecule may be represented either as a donor–acceptor model (Tl⇌P) or as a classical double bond (Tl=P).
2. Our VB theoretical analysis indicates that steric interactions between the bulky ligands coordinated to both G13 and phosphorus centers in the **G13=P-Rea** molecule—formally represented as (LB)(L**_1_**)G13=P–Ga(Cl)(LB)(L**_1_**), where (LB)(L**_1_**) denotes the CH[C(Me)NAr]**_2_** (Ar = 2,6-*i*Pr**_2_**C**_6_**H**_3_**) ligand framework—result in an increase in the ∠G13–P–Ga bond angle, causing it to exceed 90°. This structural distortion arises from spatial repulsion between the substituents and is consistent with available experimental data [32,44,45].
3. Our M06-2X-D3 computational results indicate that, from both kinetic and thermodynamic perspectives, the 1,2-addition reactions of ammonia to the imine-like **B=P-Rea**, **Al=P-Rea**, **Ga=P-Rea**, and **In=P-Rea** species are energetically favorable. In contrast, the corresponding reaction involving **Tl=P-Rea** is less favorable, as illustrated in Figure 8. This theoretical prediction aligns well with available experimental findings [30,32,45].
4. Our EDA analyses reveal that the bonding interaction between ammonia and the LB-stabilized, heavy imine-like **G13=P-Rea** molecule—characterized by a G13–P–Ga framework—in the transition state (**G13=P-TS**) follows a donor–acceptor (singlet–singlet) bonding interaction rather than an electron-sharing (triplet–triplet) bonding mode.
5. Both FMO and EDA–NOCV results indicate that the bonding in the **G13=P-TS** is primarily governed by electron donation from the lone pair on the nitrogen atom of NH_3_ into the vacant p-π* orbital on the G13 center of **G13=P-Rea**. This interaction represents the dominant bonding component. A secondary, less significant contribution arises from electron donation by a lone pair orbital on the phosphorus center of the LB-supported, heavy imine-like **G13=P-Rea** molecule to the vacant σ* orbital of the N–H bond in ammonia, as illustrated in Figure 9.
6. Our ASM analysis clearly demonstrates that the activation barrier of the 1,2-addition reaction between NH_3_ and the LB-stabilized, heavy imine-like **G13=P-Rea** molecule is significantly affected by the deformation energy of NH_3_. Specifically, as the G13 element in **G13=P-Rea** becomes heavier, its atomic radius increases, resulting in a longer G13–P bond. Consequently, NH_3_ must adopt a more distorted geometry—with an elongated H_2_N–H bond—in order to interact effectively with **G13=P-Rea**, which leads to a higher deformation energy of NH_3_ and thus a greater activation barrier for the reaction.

## Figures and Tables

**Table 1:** The chemical bonding interaction between (L)G13: (where L = CH[C(Me)NAr]_2_, Ar = 2,6-*i*Pr*_2_*C*_6_*H*_3_*) and :P–L_2_ (with L_2_ = Ga(Cl)(L)) in the **G13=P-Rea** reactant (see Scheme 1) was examined using EDA at the ZORA-M06-2X-D3/TZ2P//M06-2X-D3/def2-TZVP level of theory. The EDA calculations were carried out for both singlet (S) and triplet (T) spin states. All calculated energy values are reported in kcal/mol.

Fragments	B=P-Rea		Al=P-Rea		Ga=P-Rea		In=P-Rea		Tl=P-Rea	
	(L)B: (S) + :P–L_2_ (S)	(L)B: (T) + :P–L_2_ (T)	(L)Al: (S) + :P–L_2_ (S)	(L)Al: (T) + :P–L_2_ (T)	(L)Ga: (S) + :P–L_2_ (S)	(L)Ga: (T) + :P–L_2_ (T)	(L)In: (S) + :P–L_2_ (S)	(L)In: (T) + :P–L_2_ (T)	(L)Tl: (S) + :P–L_2_ (S)	(L)Tl: (T) + :P–L_2_ (T)
Δ*E*_INT_^(a)^	−150.1	−133.4	−124.0	−131.8	−109.6	−129.2	−94.7	−134.2	−71.7	−110.6
Δ*E*_Pauli_	402.8	255.4	388.3	153.8	393.2	148.0	326.1	174.3	300.6	205.2
Δ*E*_Elstat_^(b)^	−268.4 (48.5%)	−171.8 (44.2%)	−220.0 (43.0%)	−121.1 (42.4%)	−227.1 (45.2%)	−125.2 (45.2%)	−201.0 (47.8%)	−125.1 (40.6%)	−187.5 (50.4%)	−130.0 (41.2%)
Δ*E*_Orb_^(b)^	−277.9 (50.3%)	−210.3 (54.1%)	−286.1 (55.8%)	−158.3 (55.4%)	−269.6 (53.6%)	−145.8 (52.6%)	−213.9 (50.8%)	−177.4 (57.5%)	−178.9 (48.0%)	−179.9 (57.0%)
Δ*E*_Disper_^(b)^	−6.7 (1.2%)	−6.7 (1.7%)	−6.2 (1.2%)	−6.2 (2.2%)	−6.2 (1.2%)	−6.2 (2.2%)	−5.9 (1.4%)	−5.9 (1.9%)	−6.0 (1.6%)	−6.0 (1.9%)

^(a)^ Δ*E*_INT_ = Δ*E*_Elstat_ + Δ*E*_Pauli_ + Δ*E*_Orb_ + Δ*E*_Disper_. ^(b)^ The values in parentheses offer the percentage contribution to the total attractive interactions (Δ*E*_Elstat_ + Δ*E*_Orb_).

**Table 2:** Natural orbital-based bonding data for the G13–P interaction in **G13=P-Rea** ((L)G13=P–Ga(Cl)(L); L = CH[C(Me)NAr]_2_, Ar = 2,6-*i*Pr*_2_*C*_6_*H*_3_*) molecules, including orbital occupation, polarization, hybridization, bond length (Å), and Wiberg bond indices (WBI).

Molecule	Bond Type	Occupancy	Polarization	Hybridization	Bond Length (Å)	WBI
**B=P-Rea**	σ	1.974	B: 44.30% + P: 55.70%	B: sp^1.26^d^0.00^ P: sp^1.97^d^0.01^	1.842	1.619
	π	1.876	B: 25.13% + P: 74.87%	B: sp^99.99^d^8.42^ P: sp^1.00^d^0.00^		
**Al=P-Rea**	σ	1.961	Al: 30.57% + P: 69.43%	Al: sp^0.31^d^0.00^ P: sp^4.56^d^0.03^	2.162	1.431
	π	1.872	Al: 16.57% + P: 83.43%	Al: sp^99.99^d^11.83^ P: sp^1.00^d^0.00^		
**Ga=P-Rea**	σ	1.963	Ga: 36.55% + P: 63.45%	Ga: sp^0.21^d^0.00^ P: sp^5.89^d^0.04^	2.182	1.478
	π	1.874	Ga: 16.93% + P: 83.07%	Ga: sp^99.99^d^4.95^ P: sp^99.99^d^12.03^		
**In=P-Rea**	σ	1.939	In: 42.14% + P: 57.86%	In: sp^0.07^d^0.00^ P: sp^15.17^d^0.09^	2.360	1.371
	π	1.860	In: 14.03% + P: 85.97%	In: sp^99.99^d^3.29^ P: sp^1.00^d^0.00^		
**Tl=P-Rea**	σ	1.945	Tl: 58.68% + P: 41.32%	Tl: sp^0.03^d^0.00^ P: sp^25.80^d^0.17^	2.389	1.262

**Table 3:** Energy differences (in eV) between the frontier molecular orbitals of the imine-like **G13=P-Rea** ((L)G13=P–Ga(Cl)(L); L = CH[C(Me)NAr]_2_, Ar = 2,6-*i*Pr*_2_*C*_6_*H*_3_*) compounds and ammonia, calculated according to Scheme 1 at the M06-2X-D3/def2-TZVP level of theory.

System	Energy Difference NH_3_ (LUMO)–G13=P-Rea (HOMO-1)	Energy Difference G13=P-Rea (LUMO)–NH_3_ (HOMO)
**B=P-Rea** + **NH_3_**	8.512	7.744
**Al=P-Rea** + **NH_3_**	9.023	7.898
**Ga=P-Rea** + **NH_3_**	9.244	8.071
**In=P-Rea** + **NH_3_**	9.382	8.364
**Tl=P-Rea** + **NH_3_**	9.502	8.150

**Table 4:** EDA results of **G13=P-TS** using both singlet–singlet and triplet–triplet models at the ZORA-M06-2X-D3/TZ2P//M06-2X-D3/def2-TZVP level ^(a)^.

Fragments	B=P-TS		Al=P-TS		Ga=P-TS		In=P-TS		Tl=P-TS	
	B=P-Rea (S) + H_2_N-H (S)	B=P-Rea (T) + H_2_N-H (T)	Al=P-Rea (S) + H_2_N-H (S)	Al=P-Rea (T) + H_2_N-H (T)	Ga=P-Rea (S) + H_2_N-H (S)	Ga=P-Rea (T) + H_2_N-H (T)	In=P-Rea (S) + H_2_N-H (S)	In=P-Rea (T) + H_2_N-H (T)	Tl=P-Rea (S) + H_2_N-H (S)	Tl=P-Rea (T) + H_2_N-H (T)
Δ*E*_INT_^(b)^	−55.1	−195.3	−63.9	−193.1	−55.1	−175.4	−55.7	−162.7	−53.7	−139.9
Δ*E*_Pauli_	348.4	335.4	230.4	330.3	238.4	295.3	208.3	232.5	191.4	179.6
Δ*E*_Elstat_^(c)^	−191.4 (47.4%)	−73.7 (32.7%)	−153.9 (52.3%)	−220.0 (42.0%)	−153.5 (52.3%)	−203.0 (43.1%)	−133.9 (50.7%)	−168.7 (42.7%)	−112.2 (45.8%)	−130.6 (40.9%)
Δ*E*_Orb_^(c)^	−210.8 (52.2%)	−355.7 (67.0%)	−139.0 (47.2%)	−302.1 (57.7%)	−138.8 (47.3%)	−266.5 (56.6%)	−128.8 (48.8%)	−225.3 (57.0%)	−131.7 (53.7%)	−187.7 (58.8%)
Δ*E*_Orb (1)_^(d)^	−151.7 (71.9%)	−258.2 (72.6%)	−93.6 (67.4%)	−253.2 (83.8%)	−92.1 (66.3%)	−215.0 (80.7%)	−85.6 (66.5%)	−182.7 (81.1%)	−89.6 (68.0%)	−153.5 (81.8%)
Δ*E*_Orb (2)_^(d)^	−30.3 (14.4%)	−69.0 (19.4%)	−26.0 (18.7%)	−34.2 (11.3%)	−26.4 (19.0%)	−35.3 (13.2%)	−22.8 (17.7%)	−33.6 (14.9%)	−23.7 (18.0%)	−33.0 (17.6%)
Δ*E*_Rest_^(d)^	−28.9 (13.7%)	−28.5 (8.0%)	−19.3 (13.9%)	−14.7 (4.9%)	−20.3 (14.6%)	−16.2 (6.1%)	−20.4 (15.8%)	−9.0 (4.0%)	−18.3 (13.9%)	−1.2 (0.6%)
Δ*E*_Disper_^(c)^	−1.3 (0.3%)	−1.3 (0.2%)	−1.3 (0.4%)	−1.3 (0.3%)	−1.3 (0.4%)	−1.3 (0.3%)	−1.3 (0.5%)	−1.3 (0.3%)	−1.2 (0.5%)	−1.2 (0.4%)

^(a)^ Energy values are presented in kcal/mol for all measurements. ^(b)^ Δ*E*_INT_ = Δ*E*_Elstat_ + Δ*E*_Pauli_ + Δ*E*_Orb_ + Δ*E*_Disper_. ^(c)^ The values in parentheses represent the percentage contribution of (Δ*E*_Elstat_ + Δ*E*_Orb_ + Δ*E*_Disper_) to the total attractive interactions. ^(d)^ The values enclosed in parentheses indicate the percentage of the total orbital interactions (Δ*E*_Orb_) that they contribute. ΔE_Orb (1)_: forward bonding. ΔE_Orb (2)_: backward bonding.

**Table 5:** ASM analysis of the **G13=P-TS** for the 1,2-addition reaction of NH**_3_** to a heavy imine-like **G13=P-Rea** molecule ^(a,b)^.

Entry	B=P-TS	Al=P-TS	Ga=P-TS	In=P-TS	Tl=P-TS
Δ*E*_ACT_^(b,c)^	−1.8	3.7	7.5	12.5	23.5
Δ*E*_DEF,NH3_	42.5	49.4	53.6	58.5	71.2
∆*E*_DEF,G13=P-Rea_	9.6	9.4	9.6	9.1	6.0
Δ*E*_INT_	−53.9	−55.1	−55.7	−55.1	−53.7

^(a)^ At the level of ZORA-M06-2X-D3/TZ2P//M06-2X-D3/def2-TZVP. ^(b)^ All values are in kcal mol^−1^. ^(c)^ ΔE_ACT_ = ∆E_DEF,NH3_ + ∆E_DEF,G13=P-Rea_ + ΔE_INT_.

## Data Availability

The original contributions presented in this study are included in the article and Supplementary Material. Further inquiries can be directed to the corresponding author.

## Supplementary Materials

The following supporting information can be downloaded at: https://www.mdpi.com/article/10.3390/molecules30153222/s1, The optimized geometries calculated for all stationary points (G13=P-Rea, G13=P-TS, G13=P-Prod) using the M06-2X-D3/def2-TZVP level of theory are theory are collected in the Supporting Information.
