# Supplementary material for: Reactivity of Ammonia in 1,2-Addition to Group 13 Imine Analogues with G13–P–Ga Linkages: The Electronic Role of Group 13 Elements
Source: Molecules. 2025 Jul 31;30(15):3222. doi: 10.3390/molecules30153222 (PMC12348829; doi:10.3390/molecules30153222)
Supplement: Supplementary file 1 [file molecules-30-03222-s001.zip › molecules-3767018-supplementary.pdf]

Supporting Information

Reactivity of Ammonia in 1,2-Addition to Group 13 Imine Analogues with G13–P–Ga Linkages: The Electronic Role of Group 13 Elements

Zheng-Feng Zhang[1] and Ming-Der Su[1,2]*

[1]Department of Applied Chemistry, National Chiayi University, Chiayi 60004, Taiwan

[2]Department of Medicinal and Applied Chemistry, Kaohsiung Medical University, Kaohsiung 80708, Taiwan

*E-mail: midesu@mail.ncyu.edu.tw

(All were calculated at the M06-2X-D3/def2-TZVP level)

Table S1

B=P-Rea

Atomic Number	Coordinates (Angstroms) X	Y	Z
Cl	-1.10860900	-1.82268500	2.55860500
P	0.25687600	0.64034800	-0.39524000
Ga	-1.25480100	-0.85274900	0.52215500
B	2.08018300	0.74442800	-0.15178200
N	-2.97442400	0.20952700	0.77880400
N	-2.20446100	-2.27102400	-0.56230200
N	2.69417200	2.06068300	-0.63563600
N	3.17057500	-0.14173500	0.39341800
C	-4.16517200	-0.37320700	0.76494900
C	-4.39253800	-1.68330800	0.30980200
H	-5.41974000	-2.03205300	0.36767000
C	-3.51261500	-2.49725500	-0.41291400
C	-5.39118500	0.38838700	1.21659500
H	-5.35529100	0.53338500	2.30528200
H	-6.30134300	-0.16654100	0.96630000
H	-5.43595600	1.38746200	0.76407900
C	-4.15463800	-3.67290900	-1.11684100
H	-5.24273900	-3.63516400	-1.00395100
H	-3.78814400	-4.62513400	-0.71437300
H	-3.90152500	-3.66327400	-2.18619200
C	-2.94812500	1.62191900	1.02438500
C	-3.07091800	2.13172000	2.33289100
C	-3.13260600	3.51956200	2.49826700
H	-3.23393100	3.93279700	3.50391600
C	-3.05242600	4.37954900	1.40892700
H	-3.09456600	5.45989000	1.56067100
C	-2.91529100	3.86013600	0.12498200
H	-2.85691000	4.53790500	-0.72855100
C	-2.88207200	2.47982200	-0.09326100
C	-2.90865800	1.92819300	-1.51039900
H	-2.52821700	0.89728600	-1.47715700
C	-4.35518300	1.87272500	-2.02092600
H	-4.96518800	1.16605000	-1.43898700
H	-4.38355000	1.55041100	-3.07224700
H	-4.82563200	2.86657500	-1.95687400
C	-2.01328400	2.70430000	-2.47036000
H	-2.39321300	3.71989800	-2.66670500
H	-1.96453100	2.17943800	-3.43817100
H	-0.99511400	2.77360900	-2.06354800
C	-1.50778200	-3.19264300	-1.41496400
C	-1.20814500	-4.48854900	-0.94298900
C	-0.62577500	-5.40168700	-1.82986700
H	-0.38661500	-6.40751400	-1.47946200
C	-0.36653300	-5.05577900	-3.14999600

H	0.07432100	-5.78469000	-3.83194700
C	-0.66177100	-3.77183800	-3.59840900
H	-0.43999800	-3.50959100	-4.63238500
C	-1.22587700	-2.81639600	-2.74736900
C	-1.49337900	-4.92184600	0.48843400
H	-2.18213700	-4.19552600	0.94313200
C	-2.12458300	-6.31502200	0.58181500
H	-2.43778300	-6.51256800	1.61712600
H	-1.40561700	-7.10071500	0.30499200
H	-3.00445200	-6.42743900	-0.06868100
C	-0.20592500	-4.87111200	1.30720400
H	0.23009100	-3.86674500	1.27781400
H	0.53120400	-5.58842400	0.90968600
H	-0.40180200	-5.11421000	2.36299600
C	-1.55016400	-1.41719500	-3.25613000
H	-1.22090000	-0.70640300	-2.47784400
C	-3.05266700	-1.20859800	-3.48932700
H	-3.63764700	-1.23684900	-2.55977600
H	-3.44599100	-1.97359800	-4.17816300
H	-3.22023000	-0.22389400	-3.95140700
C	-0.79414600	-1.06376200	-4.53585500
H	-0.95096300	-0.00315000	-4.77704900
H	-1.15628200	-1.65252300	-5.39315000
H	0.28684400	-1.23251200	-4.42805000
C	4.00243800	2.32376500	-0.64406300
C	4.91677700	1.36830800	-0.19864500
H	5.97935200	1.59173800	-0.22101400
C	4.48045200	0.16557300	0.33909600
C	4.52115500	3.64741800	-1.12736000
H	3.99128600	4.48014300	-0.64692000
H	4.34923000	3.74813900	-2.20833400
H	5.59526400	3.72409500	-0.92765200
C	5.50575000	-0.78461000	0.88772500
H	6.50947700	-0.36606400	0.76076800
H	5.44646300	-1.75723200	0.37989200
H	5.32030900	-0.97817500	1.95381500
C	1.83149400	3.15167200	-1.03901500
C	1.67147700	3.44101500	-2.40388500
C	0.91912300	4.57014500	-2.74374300
H	0.77144600	4.81759300	-3.79638400
C	0.34247400	5.37075800	-1.76376500
H	-0.24703900	6.24330300	-2.05000700
C	0.51213700	5.05580000	-0.41865000
H	0.05270200	5.68377000	0.34735200
C	1.27014200	3.94870000	-0.02787500
C	2.24724800	2.55372600	-3.49633100
H	3.01537100	1.90634900	-3.04591400
C	2.90819900	3.35118700	-4.62442000
H	2.16274300	3.91467300	-5.20452100
H	3.41371100	2.66825000	-5.32233400
H	3.65098500	4.07164800	-4.24969400
C	1.15733700	1.63424800	-4.05468800

H	0.69517500	1.04676000	-3.24760400
H	1.57807500	0.94639700	-4.80393800
H	0.36906000	2.23158400	-4.54094000
C	1.49584500	3.66271900	1.44805300
H	1.95891500	2.67015600	1.54022600
C	2.45120500	4.68681800	2.07073700
H	2.03871800	5.70480600	1.99509500
H	3.43554800	4.68376700	1.57852400
H	2.60689800	4.46313200	3.13680000
C	0.17201100	3.60203900	2.20333900
H	0.33756800	3.29004700	3.24520000
H	-0.50268900	2.87764800	1.72255600
H	-0.32942300	4.58124800	2.22383400
C	2.87535100	-1.39830700	1.04326600
C	2.97487900	-2.58299500	0.28986200
C	2.85524100	-3.79351200	0.97349400
H	2.92943800	-4.73090200	0.42092200
C	2.63401700	-3.82415800	2.34772700
H	2.51823000	-4.78184500	2.85753900
C	2.56460300	-2.64126600	3.07073500
H	2.41347500	-2.67790500	4.14995000
C	2.72597300	-1.40317300	2.43900500
C	2.82264100	-0.12645700	3.26053500
H	3.38428200	0.61429800	2.66877500
C	1.43874700	0.45852300	3.53351500
H	1.51815000	1.37750700	4.13618600
H	0.81761900	-0.26941300	4.07559500
H	0.91967600	0.69538100	2.59227800
C	3.59025800	-0.33020100	4.56954000
H	3.77436000	0.64229500	5.04836300
H	4.56008000	-0.82466600	4.40752200
H	3.01450600	-0.93842900	5.28223700
C	3.25957100	-2.56340800	-1.20398500
H	3.76211100	-1.61332000	-1.44442000
C	4.19039300	-3.69729200	-1.64128000
H	3.68313300	-4.67213400	-1.59233500
H	4.49685600	-3.54563500	-2.68609300
H	5.09736300	-3.75492000	-1.01996200
C	1.95185900	-2.61070600	-1.99635000
H	1.32125400	-1.73676900	-1.77871900
H	2.15356300	-2.64231300	-3.07881500
H	1.38533900	-3.51557000	-1.72839400
C	-3.10922500	1.22866900	3.55598500
H	-3.26562800	0.19440600	3.21547800
C	-1.75109900	1.25559900	4.25770100
H	-1.72326400	0.52954100	5.08439900
H	-0.95703800	0.99392000	3.55022300
H	-1.54335500	2.26003300	4.66202900
C	-4.22286300	1.59926900	4.54018800
H	-4.28756200	0.84374700	5.33672500
H	-4.02152800	2.56664700	5.02448200
H	-5.20680900	1.67243500	4.05424800

Table S2

Al=P-Rea

Atomic Number	Coordinates (Angstroms) X	Y	Z
Cl	-1.28367400	-1.82926400	2.49710200
P	-0.14771000	0.65348500	-0.53527000
Ga	-1.60145400	-0.72053800	0.55340500
Al	1.89468800	0.66712600	0.17538500
N	-3.30621900	0.25395300	0.93800800
N	-2.53240600	-2.10620700	-0.55145100
N	2.87149500	2.19002400	-0.41841400
N	3.38064900	-0.22960800	0.91134200
C	-4.45299300	-0.41101500	1.04305600
C	-4.64634700	-1.71519900	0.55826800
H	-5.63036200	-2.14560300	0.72512500
C	-3.79369100	-2.43974200	-0.29485800
C	-5.64180100	0.26537800	1.68393800
H	-5.39449200	0.54607100	2.71839100
H	-6.51307200	-0.39783700	1.68727000
H	-5.89512100	1.19765500	1.16033600
C	-4.39374000	-3.64649700	-0.97643900
H	-5.46707000	-3.71325500	-0.77100500
H	-3.90391500	-4.56600500	-0.62923200
H	-4.23193700	-3.59345600	-2.06235300
C	-3.33554700	1.66840900	1.16277300
C	-3.19141600	2.18940200	2.46342600
C	-3.34181800	3.56666300	2.64945500
H	-3.24237300	3.98496500	3.65302200
C	-3.61403300	4.41112300	1.57786300
H	-3.73694300	5.48294700	1.74302200
C	-3.70676700	3.88715700	0.29289900
H	-3.90270500	4.55426700	-0.54936400
C	-3.56539900	2.51556000	0.05985800
C	-3.72040400	1.96854300	-1.35007100
H	-3.38783700	0.92033800	-1.33801200
C	-5.19367600	1.98036900	-1.77510800
H	-5.80691500	1.34733000	-1.11681100
H	-5.30596600	1.60355300	-2.80254000
H	-5.60127600	3.00304300	-1.74147900
C	-2.84334500	2.71651700	-2.35574000
H	-3.14611900	3.77093500	-2.45564000
H	-2.93787100	2.25464800	-3.35089400
H	-1.78553700	2.67852600	-2.05426900
C	-1.80188000	-2.88307800	-1.50912300
C	-1.29545100	-4.14902800	-1.14910900
C	-0.65237700	-4.91090900	-2.13009600
H	-0.26065000	-5.89603500	-1.86768800
C	-0.48939200	-4.42986600	-3.42322100
H	0.01562200	-5.03800300	-4.17565800

C	-0.95213000	-3.15881700	-3.74980300
H	-0.79774500	-2.77818600	-4.75964300
C	-1.61302000	-2.36382300	-2.80833900
C	-1.36098300	-4.67340800	0.27635300
H	-2.04681600	-4.03894300	0.85527700
C	-1.84560700	-6.12361500	0.36712400
H	-1.96269000	-6.41362400	1.42137200
H	-1.12139800	-6.81856900	-0.08422900
H	-2.81059300	-6.27741000	-0.13770700
C	0.02265400	-4.54120600	0.91731000
H	0.40303200	-3.51139900	0.85017900
H	0.73962500	-5.20565700	0.40845500
H	-0.00854800	-4.81049800	1.98408100
C	-2.13884800	-0.98827000	-3.18723700
H	-1.99021000	-0.33880000	-2.31290000
C	-3.63792300	-1.01630500	-3.50504300
H	-4.24112500	-1.28244900	-2.62436500
H	-3.85362900	-1.73861800	-4.30848700
H	-3.96932200	-0.02268800	-3.84453200
C	-1.35481900	-0.35203500	-4.33290000
H	-1.62593500	0.70919300	-4.42688400
H	-1.56802900	-0.83708400	-5.29847600
H	-0.27443600	-0.40683800	-4.14155700
C	4.15066300	2.44340200	-0.17333700
C	4.97628600	1.55250300	0.53672200
H	6.01159200	1.85230700	0.67644900
C	4.62597300	0.27376900	0.97790300
C	4.77925000	3.69240500	-0.72897500
H	4.13302800	4.56444500	-0.56797800
H	4.89677900	3.58629600	-1.81850000
H	5.76399300	3.86606700	-0.28231500
C	5.73543000	-0.59008700	1.52289800
H	6.68780700	-0.05105700	1.50098900
H	5.82328300	-1.50980400	0.92706700
H	5.52052700	-0.90875400	2.55224400
C	2.12036000	3.08157200	-1.26349500
C	2.22259800	2.93762700	-2.65989600
C	1.41396200	3.74749800	-3.46209300
H	1.46285000	3.64660000	-4.54794000
C	0.53980100	4.67088800	-2.89782100
H	-0.08932900	5.28902400	-3.54010100
C	0.45951600	4.79872800	-1.51500200
H	-0.23734700	5.51684700	-1.07901200
C	1.24048600	4.00515500	-0.67032800
C	3.13626200	1.90268700	-3.30179500
H	3.84057100	1.54057300	-2.53769200
C	3.96774700	2.48883600	-4.44637500
H	3.33206900	2.77578500	-5.29690300
H	4.68807600	1.74314000	-4.81313800
H	4.52625800	3.38285500	-4.13096900
C	2.32725800	0.69250700	-3.78370200
H	1.72234600	0.26175700	-2.97022200

H	2.99557800	-0.08572400	-4.18454600
H	1.63390600	0.99465600	-4.58405800
C	1.13309200	4.16604000	0.83757600
H	1.62184800	3.29899100	1.30720900
C	1.87277000	5.42448800	1.30852700
H	1.43804500	6.32218900	0.84244400
H	2.94197900	5.39644900	1.05315900
H	1.78790600	5.53347500	2.39983700
C	-0.31852200	4.18103600	1.31249900
H	-0.35793900	4.17550200	2.41217900
H	-0.86406700	3.29941800	0.94129000
H	-0.84565100	5.08550400	0.97324000
C	3.21598900	-1.61269700	1.28312900
C	3.26399900	-2.59147700	0.27462800
C	3.22502200	-3.93397900	0.66640200
H	3.27166100	-4.71347900	-0.09730200
C	3.10840000	-4.28834600	2.00371100
H	3.06903900	-5.34102200	2.28787000
C	3.02015500	-3.30134700	2.98267200
H	2.91727000	-3.59326500	4.02781100
C	3.08098600	-1.94788800	2.64607100
C	3.01213200	-0.86050800	3.70774500
H	3.67368600	-0.04016800	3.38742900
C	1.59628500	-0.28864000	3.80423900
H	1.56281900	0.55220900	4.51340600
H	0.88333300	-1.06039200	4.12795200
H	1.22445600	0.06952900	2.83114900
C	3.49640200	-1.32243700	5.08158500
H	3.55315100	-0.46360000	5.76519600
H	4.49151700	-1.78936000	5.03003800
H	2.80079800	-2.04745400	5.52912500
C	3.35979900	-2.25059400	-1.20564100
H	3.39370500	-1.15433500	-1.31214500
C	4.64731400	-2.79810900	-1.83002300
H	4.66917000	-3.89763400	-1.78891700
H	4.71366500	-2.50189800	-2.88730000
H	5.54281600	-2.42289200	-1.31292800
C	2.12274800	-2.75322300	-1.95574700
H	1.19786600	-2.30783400	-1.55407200
H	2.18561500	-2.49489400	-3.02392800
H	2.03480100	-3.84792200	-1.88092900
C	-2.82035000	1.29179400	3.62929700
H	-3.02635200	0.25101500	3.34010000
C	-1.31151600	1.39096100	3.87513000
H	-0.98911800	0.64240900	4.61347100
H	-0.75347400	1.21631500	2.94211300
H	-1.04794100	2.39535100	4.24655800
C	-3.60467300	1.59467200	4.90678500
H	-3.36394700	0.85096900	5.68020000
H	-3.35173700	2.58479900	5.31524900
H	-4.69126900	1.57019700	4.73480500

Table S3

Ga=P-Rea

Atomic Number	X	Y	Z
Cl	-1.22865000	-1.82038600	2.46201400
P	-0.20638300	0.70914400	-0.56998000
Ga	-1.61656300	-0.70720400	0.53795200
Ga	1.84057700	0.64843700	0.18326300
N	-3.32390100	0.24696100	0.95039600
N	-2.54515000	-2.09431100	-0.56450000
N	2.87860100	2.21566200	-0.43646200
N	3.38648800	-0.24438100	0.94951200
C	-4.46387100	-0.42947200	1.06270100
C	-4.65229700	-1.72946800	0.56564700
H	-5.63122200	-2.16937600	0.73780000
C	-3.80190600	-2.43923700	-0.30280400
C	-5.64875700	0.23027700	1.72731700
H	-5.38730800	0.50444200	2.76006000
H	-6.51393600	-0.44078700	1.73853100
H	-5.91849900	1.16471800	1.21594800
C	-4.39758400	-3.64401500	-0.99116100
H	-5.47131800	-3.71408700	-0.78890100
H	-3.90681400	-4.56327000	-0.64457700
H	-4.23234700	-3.58774300	-2.07628500
C	-3.36205100	1.66008300	1.18456300
C	-3.19569400	2.17554700	2.48445600
C	-3.34784100	3.55149700	2.67974100
H	-3.22923400	3.96541000	3.68308400
C	-3.64591700	4.39976800	1.61834500
H	-3.76975700	5.47025600	1.79091200
C	-3.76375500	3.88106600	0.33321900
H	-3.97958800	4.55142100	-0.50147700
C	-3.61871200	2.51168600	0.09061800
C	-3.79210500	1.97134700	-1.31987100
H	-3.44526300	0.92764000	-1.32054700
C	-5.27229400	1.96263900	-1.72000700
H	-5.86482200	1.31793700	-1.05426500
H	-5.39548600	1.58763500	-2.74693000
H	-5.69428500	2.97903000	-1.67557400
C	-2.94509800	2.73886600	-2.33684500
H	-3.26654400	3.78867600	-2.42448700
H	-3.05103400	2.28217300	-3.33309900
H	-1.88091600	2.71766500	-2.05636000
C	-1.81031200	-2.85287900	-1.53351400
C	-1.29049300	-4.11847800	-1.19164300
C	-0.63650700	-4.85720000	-2.18317700
H	-0.23446400	-5.84197500	-1.93527000
C	-0.47250400	-4.35281900	-3.46717600
H	0.04343600	-4.94248500	-4.22683800

Coordinates (Angstroms)

C	-0.94887500	-3.08216000	-3.77526400
H	-0.79503700	-2.68304900	-4.77804200
C	-1.62426800	-2.31137800	-2.82425900
C	-1.35118000	-4.66777800	0.22487600
H	-2.03575800	-4.04380900	0.81686000
C	-1.83408900	-6.12018300	0.29013500
H	-1.94851300	-6.42992400	1.33904800
H	-1.10919100	-6.80500100	-0.17532500
H	-2.79935100	-6.26719800	-0.21602000
C	0.03388400	-4.54792000	0.86549800
H	0.41335000	-3.51697000	0.82005900
H	0.75095100	-5.20150400	0.34274200
H	0.00448800	-4.83956500	1.92644400
C	-2.17299400	-0.94041500	-3.18675500
H	-2.05935200	-0.30563200	-2.29724600
C	-3.66513800	-0.99737200	-3.53164100
H	-4.27467100	-1.30203400	-2.66787700
H	-3.84938400	-1.70443800	-4.35606600
H	-4.01696100	-0.00403700	-3.85096700
C	-1.38032600	-0.26183000	-4.30157800
H	-1.68377000	0.79113300	-4.38796000
H	-1.55103500	-0.73839100	-5.27961500
H	-0.30464800	-0.28314800	-4.07906900
C	4.15321800	2.45139900	-0.17833600
C	4.96474400	1.55795900	0.55008000
H	5.99970900	1.85738800	0.69345600
C	4.62136700	0.28065100	1.00163400
C	4.80704300	3.68544800	-0.74291800
H	4.15885100	4.56345500	-0.63013300
H	4.96790700	3.54856700	-1.82360100
H	5.77495200	3.86874300	-0.26405800
C	5.74257400	-0.56950600	1.54892200
H	6.68746400	-0.01735800	1.53109600
H	5.84568100	-1.48525000	0.94933300
H	5.53002000	-0.89505500	2.57647900
C	2.13686000	3.08765300	-1.30196000
C	2.25465100	2.92258800	-2.69540000
C	1.44237200	3.70633800	-3.51951600
H	1.50497800	3.58886800	-4.60304000
C	0.54606400	4.62327500	-2.97994400
H	-0.08541900	5.22108500	-3.63896400
C	0.44586500	4.76944000	-1.60013700
H	-0.26987500	5.48055000	-1.18404300
C	1.22968900	4.00219700	-0.73416200
C	3.18472200	1.88551900	-3.30967100
H	3.88602500	1.54544000	-2.53320300
C	4.01916400	2.45751100	-4.45907000
H	3.38738200	2.72260000	-5.31959400
H	4.74941500	1.71214300	-4.80648800
H	4.56639500	3.36255300	-4.15574800
C	2.39230400	0.65715700	-3.77325800
H	1.78206400	0.23869300	-2.95729400

H	3.07206400	-0.12406800	-4.14806400
H	1.70562600	0.93379500	-4.58849100
C	1.10307300	4.17936100	0.77055300
H	1.56113400	3.30263500	1.25260700
C	1.87411700	5.41910400	1.24077400
H	1.47499900	6.32494600	0.75874500
H	2.94537100	5.35352500	1.00149700
H	1.77716500	5.54183600	2.32965200
C	-0.35233200	4.23713700	1.22998400
H	-0.40174400	4.24234800	2.32925300
H	-0.91646100	3.36543000	0.86337500
H	-0.85510300	5.15140300	0.87928200
C	3.23415300	-1.62447400	1.32560300
C	3.28471100	-2.60798200	0.32119300
C	3.23670000	-3.94906300	0.71687300
H	3.28486100	-4.73110600	-0.04415500
C	3.10961100	-4.29911300	2.05433100
H	3.06259400	-5.35070500	2.34136800
C	3.02473300	-3.30827400	3.02977600
H	2.91693100	-3.59605700	4.07565500
C	3.09472000	-1.95627400	2.68931700
C	3.02932100	-0.86645000	3.74854400
H	3.68191500	-0.04296700	3.41906000
C	1.61137100	-0.30246700	3.85710400
H	1.57960300	0.53701700	4.56798800
H	0.90533200	-1.07852400	4.18610700
H	1.23220100	0.05587100	2.88745400
C	3.53027500	-1.32213400	5.11863200
H	3.59129200	-0.46097500	5.79901800
H	4.52657100	-1.78530600	5.05732200
H	2.84289100	-2.04862500	5.57648000
C	3.39084600	-2.27314800	-1.15993100
H	3.44648800	-1.17836300	-1.26980300
C	4.66913500	-2.84766800	-1.77932400
H	4.66716700	-3.94749500	-1.74131400
H	4.74728900	-2.55041300	-2.83550700
H	5.56977600	-2.49343100	-1.25658000
C	2.14784400	-2.75708700	-1.91325700
H	1.22746000	-2.29081400	-1.52458700
H	2.22267500	-2.51252400	-2.98384600
H	2.03637600	-3.84876900	-1.82605900
C	-2.80059200	1.27545700	3.64039100
H	-2.99599500	0.23386200	3.34655100
C	-1.29118400	1.39400500	3.87294700
H	-0.95288700	0.65062000	4.60929800
H	-0.73914400	1.22493900	2.93544300
H	-1.03668200	2.40193800	4.24082600
C	-3.57710500	1.56024400	4.92696800
H	-3.31833800	0.81627100	5.69427200
H	-3.33475500	2.55195800	5.33792500
H	-4.66479200	1.52093300	4.76510400

Table S4

In=P-Rea

Atomic Number	X	Y	Z
Cl	-1.03720100	-1.65388600	2.25647700
P	-0.42370800	0.83664900	-0.81468700
Ga	-1.73471800	-0.61416400	0.36903500
In	1.69425700	0.55749700	0.18843100
N	-3.43929500	0.25018000	0.91599700
N	-2.65272800	-2.07610500	-0.62913400
N	2.95823900	2.27571300	-0.43658400
N	3.48608400	-0.30017300	1.01206800
C	-4.55363100	-0.45807100	1.07430100
C	-4.72965200	-1.75741500	0.57033400
H	-5.68991900	-2.22301400	0.77731500
C	-3.88932000	-2.45170000	-0.32270900
C	-5.71607300	0.16469400	1.80888500
H	-5.40624000	0.42117000	2.83285400
H	-6.56874200	-0.52118400	1.84885400
H	-6.02511300	1.10528600	1.33222400
C	-4.46782600	-3.68438700	-0.97359700
H	-5.53987700	-3.76632500	-0.76662100
H	-3.96176300	-4.58259600	-0.59551300
H	-4.30369200	-3.66199000	-2.05984700
C	-3.48080200	1.66098000	1.16220600
C	-3.23266100	2.17564000	2.44832300
C	-3.36214200	3.55371200	2.64921600
H	-3.17960400	3.96793100	3.64310900
C	-3.70871700	4.40357100	1.60471200
H	-3.80981800	5.47570700	1.78129400
C	-3.90106400	3.88504400	0.32768900
H	-4.14902800	4.55809100	-0.49534500
C	-3.78453000	2.51426700	0.08054800
C	-4.02375600	1.96767800	-1.31840400
H	-3.61686000	0.94617100	-1.34804300
C	-5.52411200	1.86512700	-1.61773600
H	-6.02694200	1.17636800	-0.92327300
H	-5.69117500	1.49179800	-2.63916700
H	-6.00581200	2.85179900	-1.53088500
C	-3.29919300	2.78069500	-2.39258900
H	-3.71340400	3.79675500	-2.48378100
H	-3.41791300	2.29298200	-3.37239300
H	-2.22373000	2.85593300	-2.17050200
C	-1.90349700	-2.82134200	-1.59839300
C	-1.31795500	-4.05579900	-1.24754300
C	-0.62422400	-4.76469300	-2.23475500
H	-0.17161900	-5.72553800	-1.97940200
C	-0.48076900	-4.25970300	-3.52037700
H	0.06921900	-4.82521000	-4.27444500

C	-1.02474400	-3.01855500	-3.83792900
H	-0.88954400	-2.61717000	-4.84242700
C	-1.74391900	-2.28015400	-2.89422400
C	-1.34628400	-4.61004300	0.16889800
H	-2.01522700	-3.98517400	0.77823400
C	-1.83037200	-6.06309400	0.23476800
H	-1.91659900	-6.38284600	1.28345000
H	-1.11664700	-6.74161600	-0.25651600
H	-2.80719200	-6.20925200	-0.24795100
C	0.05316400	-4.50712300	0.78269600
H	0.44565400	-3.48240600	0.72865300
H	0.75255300	-5.17412500	0.25265200
H	0.03809000	-4.79617600	1.84466200
C	-2.36692200	-0.94529200	-3.27137400
H	-2.31431800	-0.30595200	-2.38047800
C	-3.84653900	-1.09425800	-3.64125000
H	-4.44459600	-1.45357500	-2.79059300
H	-3.97296900	-1.79945100	-4.47790200
H	-4.25954500	-0.12121900	-3.95002000
C	-1.59393500	-0.21520100	-4.36772900
H	-1.97520600	0.81061700	-4.47175600
H	-1.69946800	-0.70799500	-5.34675800
H	-0.52666600	-0.15122200	-4.11137700
C	4.23087700	2.46446600	-0.16540100
C	5.02306400	1.54759200	0.56992900
H	6.05960200	1.84403100	0.71119100
C	4.70090900	0.26750800	1.03307300
C	4.93578600	3.67897800	-0.72098700
H	4.29076300	4.56589300	-0.68143500
H	5.17503600	3.50478400	-1.78193100
H	5.87042000	3.87100000	-0.18230000
C	5.84833900	-0.55563900	1.57541700
H	6.78045400	0.01781800	1.55972900
H	5.97337700	-1.46481300	0.96947000
H	5.64538500	-0.89306400	2.60135400
C	2.23769200	3.14491900	-1.31092300
C	2.37792100	2.99163500	-2.70538500
C	1.57232200	3.77127000	-3.53982200
H	1.65887600	3.66226600	-4.62275300
C	0.64774400	4.66688600	-3.01357500
H	0.02252300	5.26191000	-3.68092300
C	0.50566000	4.78728800	-1.63483800
H	-0.24151600	5.47095700	-1.22960700
C	1.28434200	4.02613800	-0.75979500
C	3.30700400	1.95011000	-3.31323000
H	3.98634800	1.58896000	-2.52719000
C	4.17219800	2.52169900	-4.43970800
H	3.56070300	2.81150500	-5.30700300
H	4.89507300	1.76833300	-4.78555000
H	4.72981800	3.41183400	-4.11264500
C	2.50063900	0.74183300	-3.80822300
H	1.86725800	0.32020300	-3.01172800

H	3.17148700	-0.04637100	-4.18399600
H	1.82958200	1.04093300	-4.62856100
C	1.10670100	4.15956400	0.74420100
H	1.45933400	3.22307500	1.20474400
C	1.98408800	5.28799800	1.29860300
H	1.70803600	6.24928100	0.83827500
H	3.05017300	5.10840300	1.09663200
H	1.85517000	5.37943000	2.38735400
C	-0.35312300	4.33337800	1.15747600
H	-0.44136500	4.29145900	2.25341500
H	-0.97951900	3.53294400	0.73237800
H	-0.76013900	5.30530700	0.83814800
C	3.35612600	-1.67064300	1.41253600
C	3.39056400	-2.67719400	0.42776100
C	3.28703700	-4.00898800	0.84333800
H	3.31816300	-4.80473200	0.09566900
C	3.12662900	-4.33292000	2.18392100
H	3.03589800	-5.37709500	2.48698100
C	3.06914300	-3.32278200	3.14145800
H	2.93793000	-3.58840100	4.19085100
C	3.18990900	-1.97992600	2.77981400
C	3.13664900	-0.87357700	3.82281700
H	3.76953900	-0.04778700	3.46388600
C	1.71490300	-0.32465300	3.96312400
H	1.68794900	0.50834800	4.68179700
H	1.02170000	-1.11031600	4.29857800
H	1.31055400	0.04018000	3.00597700
C	3.67816600	-1.30415800	5.18621200
H	3.75251000	-0.43262900	5.85206000
H	4.67553900	-1.76097500	5.10216800
H	3.01078100	-2.02932400	5.67485000
C	3.52294200	-2.36884300	-1.05742800
H	3.66481500	-1.28243400	-1.17447800
C	4.75421700	-3.04482600	-1.66946700
H	4.66348200	-4.14091300	-1.63392000
H	4.86354100	-2.75393700	-2.72453600
H	5.67582700	-2.76515600	-1.13870600
C	2.25025400	-2.77078300	-1.81326900
H	1.35487000	-2.24463600	-1.44110600
H	2.34630200	-2.54463700	-2.88611300
H	2.06165500	-3.85067100	-1.71190700
C	-2.76668800	1.28060100	3.58212600
H	-2.92267700	0.23468800	3.28017800
C	-1.25859500	1.46610700	3.78315500
H	-0.87068400	0.73063500	4.50298200
H	-0.72148400	1.33046500	2.83140000
H	-1.04134600	2.47972500	4.15865900
C	-3.52439000	1.51894300	4.88983100
H	-3.20491200	0.79054900	5.64924700
H	-3.32822200	2.52328200	5.29474000
H	-4.61187100	1.41906900	4.75617600

Table S5

Tl=P-Rea

Atomic Number	Coordinates (Angstroms) X	Y	Z
Cl	-0.95756900	-1.59190900	2.18022800
P	-0.47417700	0.88959700	-0.85851800
Ga	-1.77877200	-0.58072100	0.33061300
Tl	1.60988000	0.44323300	0.22189000
N	-3.47826000	0.25121300	0.91033400
N	-2.67331400	-2.05629000	-0.65419200
N	2.95411700	2.29070500	-0.44645700
N	3.51609600	-0.30249600	1.05301900
C	-4.58525000	-0.47061100	1.06659000
C	-4.75052400	-1.76648200	0.55082700
H	-5.70495300	-2.24379200	0.75813900
C	-3.90410400	-2.44862900	-0.34693500
C	-5.74787200	0.13473700	1.81429800
H	-5.43146000	0.39055400	2.83640600
H	-6.59239500	-0.56080000	1.85856100
H	-6.07141300	1.07399400	1.34465900
C	-4.46520400	-3.68792900	-0.99876300
H	-5.53728600	-3.78249000	-0.79748900
H	-3.95018900	-4.57836200	-0.61430700
H	-4.29438900	-3.66667500	-2.08394000
C	-3.52802600	1.66011200	1.16998800
C	-3.25826500	2.16596000	2.45503800
C	-3.39217400	3.54170100	2.66870500
H	-3.19237100	3.94946300	3.66192400
C	-3.76254300	4.39759200	1.63746300
H	-3.86608800	5.46767500	1.82414900
C	-3.97315500	3.88824200	0.35963600
H	-4.23720000	4.56665000	-0.45397100
C	-3.85239900	2.52019900	0.09980900
C	-4.09972000	1.98589400	-1.30269900
H	-3.70135700	0.96097200	-1.34240000
C	-5.60070500	1.89511200	-1.60168700
H	-6.10830900	1.20857100	-0.90861200
H	-5.77063200	1.52584900	-2.62412700
H	-6.07479300	2.88509500	-1.51189600
C	-3.37180000	2.80361300	-2.37170600
H	-3.77912400	3.82331300	-2.45100500
H	-3.49660200	2.32695100	-3.35604100
H	-2.29477500	2.87444900	-2.15381900
C	-1.90876600	-2.78043300	-1.62820400
C	-1.29131200	-4.00125200	-1.28462900
C	-0.57570500	-4.68261100	-2.27589100
H	-0.09806500	-5.63291500	-2.02681900
C	-0.44112000	-4.16308700	-3.55634400
H	0.12699600	-4.70700200	-4.31276500

C	-1.01691400	-2.93416700	-3.86586800
H	-0.88742000	-2.52058400	-4.86604200
C	-1.75885200	-2.22340400	-2.91895900
C	-1.30837100	-4.57224300	0.12561500
H	-1.97748300	-3.95758700	0.74541800
C	-1.78591900	-6.02853700	0.17400500
H	-1.86302700	-6.36419500	1.21839500
H	-1.07165600	-6.69527200	-0.33232900
H	-2.76476600	-6.17375500	-0.30453500
C	0.09392700	-4.47540900	0.73540700
H	0.48731500	-3.45073400	0.69221600
H	0.79213600	-5.13664800	0.19679400
H	0.08218700	-4.77736800	1.79371500
C	-2.41298200	-0.90078700	-3.28807500
H	-2.38293600	-0.26828700	-2.39064700
C	-3.88724400	-1.08186900	-3.66506100
H	-4.47834300	-1.46956900	-2.82213500
H	-3.99230500	-1.77783300	-4.51215800
H	-4.32461900	-0.11553500	-3.96104300
C	-1.65266900	-0.14199700	-4.37393000
H	-2.06332100	0.87245800	-4.47703000
H	-1.73660600	-0.63265100	-5.35591500
H	-0.58988200	-0.04792300	-4.10811400
C	4.22001500	2.48259300	-0.19118800
C	5.01887300	1.57374000	0.56197300
H	6.05461900	1.87914700	0.69275200
C	4.71674800	0.30143600	1.04629200
C	4.93843400	3.67933200	-0.77420800
H	4.27057700	4.54573800	-0.85966800
H	5.28521300	3.43204700	-1.79045400
H	5.81534300	3.94129100	-0.17032000
C	5.88244700	-0.49455400	1.59657500
H	6.80258400	0.09791500	1.57560500
H	6.02757700	-1.40585700	0.99822100
H	5.68758000	-0.82709800	2.62574000
C	2.21044700	3.11720700	-1.32813300
C	2.33391700	2.93995700	-2.72211200
C	1.48648200	3.66907600	-3.56120000
H	1.56194100	3.53852500	-4.64283200
C	0.53544500	4.54039300	-3.04166600
H	-0.12183200	5.09581500	-3.71246100
C	0.40800800	4.68501700	-1.66306900
H	-0.36239600	5.34487500	-1.26214500
C	1.22770400	3.97355600	-0.78492200
C	3.28929600	1.91735700	-3.32017400
H	3.98035300	1.58585300	-2.53183400
C	4.13326500	2.49893400	-4.45741200
H	3.50929700	2.76605400	-5.32331400
H	4.87337200	1.76131100	-4.80119600
H	4.66902600	3.40570200	-4.14007900
C	2.51577500	0.67987000	-3.79437200
H	1.89647000	0.25416600	-2.98892800

H	3.20666600	-0.09636800	-4.15872400
H	1.83479300	0.94615700	-4.61791500
C	1.06928400	4.11306400	0.72006200
H	1.37565600	3.15430600	1.16877700
C	2.01500100	5.18594400	1.27155700
H	1.78791400	6.16474700	0.82141800
H	3.06589300	4.94622600	1.05323300
H	1.90637800	5.27575600	2.36277800
C	-0.37355100	4.36198000	1.15140100
H	-0.45153500	4.31777600	2.24805400
H	-1.04562900	3.59742000	0.72967300
H	-0.73295100	5.35595000	0.84212200
C	3.41600700	-1.66936600	1.46061700
C	3.45854800	-2.68298300	0.48182900
C	3.35376300	-4.01330800	0.90140400
H	3.39058500	-4.81223400	0.15717700
C	3.18740400	-4.33243500	2.24275700
H	3.09660800	-5.37544700	2.54989000
C	3.12824700	-3.31798000	3.19566500
H	2.99541800	-3.57906500	4.24620200
C	3.24762400	-1.97626500	2.82877600
C	3.18784800	-0.86780800	3.86901100
H	3.79414800	-0.02943200	3.49471000
C	1.75680600	-0.34974900	4.03354400
H	1.72303100	0.47147900	4.76548500
H	1.08414900	-1.15397100	4.36817800
H	1.33795300	0.02328300	3.08673000
C	3.76441300	-1.28327100	5.22293200
H	3.83437700	-0.40855100	5.88517100
H	4.76899100	-1.71991500	5.11994600
H	3.12231400	-2.02083000	5.72691400
C	3.59947400	-2.37810300	-1.00331400
H	3.74051800	-1.29198700	-1.12032400
C	4.83454300	-3.05525400	-1.60634800
H	4.74442800	-4.15141000	-1.56895300
H	4.95123800	-2.76622200	-2.66119800
H	5.75194200	-2.77374300	-1.06957400
C	2.33347200	-2.78413800	-1.76838200
H	1.43019900	-2.26793700	-1.40065500
H	2.43346900	-2.55440700	-2.83997700
H	2.14924700	-3.86523100	-1.67043100
C	-2.76081400	1.26729500	3.57245200
H	-2.90007400	0.22205200	3.26024400
C	-1.25440400	1.48221200	3.75891600
H	-0.84251100	0.74592600	4.46435800
H	-0.72442600	1.37119500	2.79988700
H	-1.05410600	2.49492800	4.14552500
C	-3.50832500	1.47552600	4.89120000
H	-3.16186500	0.74895800	5.64040900
H	-3.33190800	2.48109000	5.30198500
H	-4.59451700	1.34990100	4.76967200

Table S6

NH_3

Atomic Number	X	Y	Z
N	-3.91279600	-0.39370800	-0.02247300
H	-2.89572700	-0.44843200	0.01177500
H	-4.22140500	-0.41461600	0.94857700
H	-4.22138200	-1.27660500	-0.42731900

Table S7

B=P-TS

Atomic Number	X	Y	Z
Ga	0.02619300	-0.09430700	0.23849800
B	-0.12060000	-0.01380600	3.94539400
Cl	2.31093900	-0.10796900	0.16776800
N	1.12974500	0.84978900	3.52104300
H	1.78224600	0.36180600	2.90143800
H	1.64379000	1.38707000	4.22463400
P	-1.15226400	0.14453600	2.20454700
H	0.20536800	1.22669100	2.65546700
N	-0.42794100	-1.35472000	-1.25138300
N	-0.28147700	1.52076300	-0.94367000
N	-0.92584400	0.87193100	5.01065400
N	0.26338700	-1.35197600	4.69298300
C	-0.23363200	-1.02648300	-2.53020600
C	0.01265700	0.27133100	-2.98787200
H	0.16854800	0.37938900	-4.05775700
C	-0.13902500	1.46645800	-2.26092500
C	-0.34828500	-2.09787300	-3.58809600
H	-1.37246400	-2.49744500	-3.61246200
H	0.31141300	-2.94478800	-3.35887100
H	-0.09777500	-1.69512900	-4.57473600
C	-0.17546300	2.73647400	-3.08016200
H	-1.04377400	3.35318000	-2.81066800
H	-0.21179600	2.50204100	-4.14904600
H	0.71746400	3.34368400	-2.87989400
C	-0.88452500	-2.68758700	-0.99358900
C	0.05426300	-3.72242500	-0.80796900
C	-0.42342400	-5.01520500	-0.56760200
H	0.29077800	-5.82500300	-0.40177700
C	-1.78777900	-5.28969500	-0.57208900
H	-2.14239300	-6.30691900	-0.39732600
C	-2.69904300	-4.26594400	-0.81430800
H	-3.76841100	-4.48800800	-0.82786100
C	-2.26963000	-2.95160400	-1.02024800
C	1.54771100	-3.46158300	-0.92039800

H	1.68575600	-2.44416900	-1.31052500
C	2.23714900	-4.43723100	-1.87998600
H	2.20971100	-5.46728400	-1.49324400
H	3.29432500	-4.15887300	-2.00055800
H	1.77082100	-4.44306900	-2.87615600
C	2.21885800	-3.51228100	0.44985900
H	1.67836700	-2.90008000	1.18817200
H	3.25123400	-3.13581800	0.38893000
H	2.24181300	-4.54701800	0.82026000
C	-3.28910900	-1.85004200	-1.26740400
H	-2.74245200	-0.94588600	-1.57566500
C	-4.26791400	-2.20609800	-2.38978500
H	-4.90817700	-3.05658300	-2.11065500
H	-3.74375100	-2.47001600	-3.32000800
H	-4.92814000	-1.35184200	-2.60095600
C	-4.03730700	-1.51281100	0.02531100
H	-4.75208600	-0.69286800	-0.14674800
H	-3.33486800	-1.20303800	0.81481300
H	-4.60385100	-2.38636400	0.38713900
C	-0.56083100	2.80537800	-0.37501700
C	0.46719500	3.76268000	-0.24035100
C	0.11409800	5.05394900	0.16234400
H	0.89039900	5.81660700	0.25341800
C	-1.20348200	5.37731600	0.46840300
H	-1.45878600	6.39105300	0.78297900
C	-2.19138100	4.39959800	0.40218600
H	-3.21672300	4.65291300	0.67435300
C	-1.89433900	3.10294800	-0.03005300
C	1.92981800	3.41828300	-0.48279600
H	1.97544300	2.45750400	-1.01532800
C	2.67098300	4.47190500	-1.31168300
H	2.15608400	4.70402600	-2.25530800
H	3.68251900	4.11490800	-1.55365400
H	2.78277900	5.41480900	-0.75543300
C	2.65101200	3.21340900	0.85467300
H	2.71005400	4.16605000	1.40528800
H	3.67171900	2.83972400	0.68476600
H	2.12539700	2.48001000	1.48112700
C	-2.99611600	2.06682100	-0.19741600
H	-2.54059900	1.07554400	-0.06087300
C	-3.58935300	2.10650600	-1.61028100
H	-4.39373500	1.35995400	-1.70617300
H	-2.83262800	1.88040600	-2.37643200
H	-4.01626500	3.09823000	-1.82882700
C	-4.07630500	2.19435100	0.87133200
H	-4.77733000	1.34905900	0.80400800
H	-4.66673400	3.11784500	0.76138600
H	-3.61807300	2.17909700	1.86960100
C	-0.70347600	0.74919000	6.32059000
C	0.02786000	-0.32068300	6.83175000
H	0.15528800	-0.40572000	7.90688000
C	0.35820200	-1.41080700	6.02308200

C	-1.29255400	1.72862000	7.30019500
H	-2.38294900	1.79610900	7.19241900
H	-0.89700400	2.73485900	7.11004900
H	-1.04326400	1.43136100	8.32435200
C	0.77028500	-2.67966900	6.72275500
H	0.55672900	-2.59668100	7.79378000
H	1.84069700	-2.87212500	6.58820200
H	0.24169800	-3.54769200	6.30749100
C	-1.95808400	1.82480400	4.65774300
C	-1.65362300	3.19138300	4.53624100
C	-2.70304200	4.08614500	4.29586500
H	-2.47964500	5.14778400	4.17329200
C	-4.02037500	3.64992800	4.23480700
H	-4.82723900	4.36370200	4.06162200
C	-4.30622200	2.29742900	4.39706400
H	-5.34246900	1.95649300	4.35347500
C	-3.28818500	1.36327600	4.59830000
C	-0.23824600	3.72654000	4.66377200
H	0.38996700	2.91466600	5.05861400
C	-0.13150700	4.91201000	5.63012400
H	-0.66741800	5.78880200	5.23846300
H	0.92217400	5.20263700	5.75331700
H	-0.54424600	4.69003300	6.62478000
C	0.29743000	4.13268200	3.29150400
H	-0.21595900	5.04163100	2.94732700
H	0.11690500	3.35648200	2.53353300
H	1.37606000	4.34699600	3.33550100
C	-3.64011200	-0.10267100	4.79253400
H	-2.70769300	-0.67843600	4.72361200
C	-4.23892200	-0.35066700	6.18221600
H	-5.14030400	0.26304000	6.33615200
H	-3.52302200	-0.11264400	6.98292500
H	-4.52443000	-1.40731900	6.29446400
C	-4.57157200	-0.61035900	3.69115700
H	-4.71835400	-1.69695400	3.79066000
H	-4.13636000	-0.40430100	2.70310500
H	-5.56504800	-0.13910400	3.74868500
C	0.67423700	-2.56045000	4.01406200
C	2.04470400	-2.90418500	3.99765100
C	2.39108200	-4.20053000	3.60768200
H	3.44284100	-4.49300200	3.59975000
C	1.42271400	-5.11752600	3.21654200
H	1.71475500	-6.12588900	2.91767300
C	0.08610700	-4.73867700	3.17232600
H	-0.66523000	-5.45322200	2.83505200
C	-0.31606600	-3.46461700	3.58790700
C	3.15421100	-1.92890800	4.37338300
H	2.69167800	-1.04910200	4.84703200
C	4.18889600	-2.50735700	5.34644900
H	3.74870200	-2.89708100	6.27420000
H	4.91378400	-1.72760100	5.62164400
H	4.75623300	-3.32511100	4.87858700

C	3.87997800	-1.45866300	3.10715900
H	3.19068100	-1.09540000	2.33420900
H	4.44044200	-2.29595800	2.66175200
H	4.59962100	-0.65987600	3.34380200
C	-1.79122400	-3.11455700	3.67588800
H	-1.87022700	-2.03174400	3.51540200
C	-2.36971900	-3.44621900	5.05626100
H	-3.45316300	-3.25070000	5.06561600
H	-1.92001400	-2.83674200	5.85503200
H	-2.21645600	-4.50935100	5.30204800
C	-2.60373800	-3.76823500	2.56500300
H	-2.69145400	-4.85906300	2.69095200
H	-2.14197900	-3.56668300	1.58958800
H	-3.62275600	-3.35471200	2.55225000

Table S8

Al=P-TS

Atomic Number	Coordinates (Angstroms) X	Y	Z
Ga	-0.02039600	0.02493800	0.02656000
Al	-0.09536200	-0.11406600	3.82606100
Cl	2.26396000	-0.04336300	0.11524500
N	1.28450800	1.05201900	3.24501600
H	1.97670600	0.68982800	2.58333300
H	1.69221000	1.82889200	3.76507200
P	-1.27179900	0.22183300	1.91270400
H	0.17499000	1.20483300	2.41598500
N	-0.45734400	-1.28206500	-1.39002400
N	-0.30107600	1.60394200	-1.17645600
N	-1.06763800	0.85549900	5.17833700
N	0.43598600	-1.56203300	4.93947000
C	-0.25647600	-1.00624800	-2.67766800
C	-0.01207300	0.28074000	-3.17702700
H	0.15584700	0.35265800	-4.24847300
C	-0.15290500	1.50436300	-2.49039900
C	-0.33356800	-2.13151500	-3.67945300
H	-1.32701800	-2.60087700	-3.65596800
H	0.38856800	-2.91729700	-3.41671600
H	-0.12785400	-1.76949800	-4.69198100
C	-0.15951700	2.75533900	-3.33520500
H	-1.02876500	3.38278800	-3.09428300
H	-0.17225300	2.50533600	-4.40111800
H	0.73341700	3.35748200	-3.11919800
C	-0.95404300	-2.57683600	-1.03216000
C	-0.05496000	-3.62917500	-0.77005800
C	-0.57713400	-4.88388400	-0.43959500
H	0.10858900	-5.70712500	-0.22694900
C	-1.95038400	-5.09999200	-0.39048000
H	-2.34110600	-6.08870400	-0.14311400
C	-2.82479900	-4.04899600	-0.64704400

H	-3.90284900	-4.21953600	-0.59828500
C	-2.34882500	-2.77266600	-0.96207100
C	1.44771400	-3.41942800	-0.81932200
H	1.64292100	-2.42594100	-1.24582200
C	2.16068500	-4.45968500	-1.68832600
H	2.06875200	-5.47038300	-1.26247500
H	3.23325400	-4.22497300	-1.75169100
H	1.75745500	-4.49111000	-2.71134900
C	2.01981500	-3.43044600	0.59925000
H	1.46356900	-2.75054400	1.26523700
H	3.07378000	-3.11464200	0.59640000
H	1.95410100	-4.44204000	1.02425200
C	-3.33863600	-1.65140500	-1.23872600
H	-2.76451300	-0.73108600	-1.42371700
C	-4.17269700	-1.93933900	-2.49178300
H	-4.78129600	-2.84736200	-2.36012200
H	-3.53830000	-2.08443700	-3.37810700
H	-4.85666300	-1.10267800	-2.69829400
C	-4.23834900	-1.39667700	-0.02669500
H	-4.94297600	-0.57856600	-0.24282500
H	-3.63567700	-1.11427400	0.84979200
H	-4.83254400	-2.28954800	0.22545700
C	-0.56986700	2.89094200	-0.60285300
C	0.47510800	3.81735700	-0.40391200
C	0.15185700	5.08572800	0.09042400
H	0.94863000	5.81765400	0.24035400
C	-1.15742700	5.42148600	0.41042100
H	-1.39002000	6.41581400	0.79617800
C	-2.16835100	4.47656900	0.26351600
H	-3.18846000	4.73639500	0.54700900
C	-1.90047500	3.20249700	-0.24577300
C	1.93600400	3.47068900	-0.64789000
H	1.98276100	2.49459900	-1.15191700
C	2.66474500	4.50538500	-1.51173100
H	2.15835000	4.68661600	-2.47058800
H	3.68757400	4.16279900	-1.72602100
H	2.74479500	5.47277800	-0.99333300
C	2.65819300	3.31645700	0.69571500
H	2.72354400	4.28979800	1.20915100
H	3.67706900	2.92981400	0.54489300
H	2.12105500	2.61681100	1.34910200
C	-3.02832000	2.20036300	-0.44446300
H	-2.60078800	1.19833500	-0.30326600
C	-3.59628500	2.26106000	-1.86662400
H	-4.42584800	1.54440100	-1.97409800
H	-2.83680200	2.00372200	-2.61992800
H	-3.98272200	3.26720700	-2.09417900
C	-4.12865800	2.34758300	0.60380200
H	-4.84197700	1.51500600	0.51600400
H	-4.70131900	3.28018100	0.47906000
H	-3.69775000	2.32420200	1.61573700
C	-0.82977600	0.68357000	6.47572700

C	-0.03408400	-0.35971700	6.98437000
H	0.08532000	-0.39119100	8.06404900
C	0.45392100	-1.46545200	6.27289000
C	-1.46880100	1.61025100	7.47797200
H	-2.56193900	1.60412600	7.36298700
H	-1.14411900	2.64333200	7.29516000
H	-1.20654600	1.32120300	8.50088100
C	0.99288600	-2.61391000	7.08735600
H	0.87724100	-2.41680500	8.15775200
H	2.05427300	-2.78346200	6.86199400
H	0.46745500	-3.54380700	6.82727000
C	-2.06654500	1.82500000	4.80059100
C	-1.72699700	3.18171300	4.65985100
C	-2.74145500	4.09424200	4.34632100
H	-2.48984700	5.15004400	4.22525000
C	-4.05452000	3.67744200	4.18073800
H	-4.83542400	4.40196000	3.94462000
C	-4.36630300	2.32373000	4.28174100
H	-5.39177400	1.99915400	4.10424700
C	-3.38506700	1.37378800	4.57163700
C	-0.29619600	3.68163300	4.76349900
H	0.32841300	2.84135600	5.10251100
C	-0.12937900	4.82388100	5.77134700
H	-0.70444100	5.70900100	5.46169000
H	0.92722000	5.12331000	5.83384600
H	-0.46399400	4.54638300	6.78105700
C	0.18697800	4.13145400	3.37824400
H	-0.31844600	5.06467400	3.08785800
H	-0.04231000	3.38736700	2.59987300
H	1.27166600	4.32527300	3.37762700
C	-3.72896600	-0.10829500	4.60978400
H	-2.85288900	-0.63998000	4.21059300
C	-3.98640100	-0.61786100	6.03244500
H	-4.79552400	-0.04308400	6.51040300
H	-3.09099700	-0.54969700	6.66672600
H	-4.29096900	-1.67517200	6.00770700
C	-4.89669100	-0.46012800	3.68902200
H	-4.98436500	-1.55294200	3.59856100
H	-4.73793400	-0.04247700	2.68396400
H	-5.85625000	-0.08802900	4.08018000
C	0.84172200	-2.81434900	4.35734800
C	2.21365200	-3.11886300	4.22686500
C	2.56591300	-4.39728100	3.78915100
H	3.62176700	-4.65778600	3.69275900
C	1.59457600	-5.33818500	3.45812400
H	1.89108700	-6.33248400	3.11977400
C	0.24951300	-4.99623200	3.52326500
H	-0.51063200	-5.72309700	3.22833500
C	-0.15300200	-3.73487200	3.97713600
C	3.29622600	-2.08546800	4.49856000
H	2.83340800	-1.23900900	5.02784700
C	4.44024500	-2.61980500	5.36504300

H	4.08411600	-3.04535600	6.31454800
H	5.14622500	-1.80964400	5.59832000
H	5.00597200	-3.40339800	4.83976000
C	3.84475400	-1.55526400	3.16829600
H	3.04410100	-1.22577600	2.49120100
H	4.40229200	-2.34730700	2.64354300
H	4.53090500	-0.71124500	3.33680700
C	-1.63433800	-3.41016800	4.06823200
H	-1.73257000	-2.36890300	4.40508200
C	-2.33750100	-4.28838400	5.10818900
H	-3.40662300	-4.03414700	5.16339400
H	-1.90294100	-4.15635800	6.11044500
H	-2.25947700	-5.35379000	4.84226400
C	-2.30336900	-3.52265600	2.69694800
H	-2.32369900	-4.56699600	2.35038300
H	-1.77223200	-2.92018400	1.94349900
H	-3.34338800	-3.16338000	2.74441500

Table S9

Ga=P-TS

Atomic Number	Coordinates (Angstroms) X	Y	Z
Ga	-0.01053900	0.05476700	0.00074200
Ga	-0.11159000	-0.13001900	3.78876100
Cl	2.26423400	-0.05571100	0.11130600
N	1.28371200	1.14513900	3.21108700
H	1.99747200	0.76500000	2.58371300
H	1.70538900	1.87493100	3.78681500
P	-1.27390000	0.38132000	1.87080600
H	0.15370000	1.32029200	2.35047700
N	-0.46394900	-1.27155900	-1.39036300
N	-0.27919000	1.62050000	-1.21776300
N	-1.10715000	0.85477400	5.21160400
N	0.47351800	-1.58621200	4.96169600
C	-0.27097500	-1.01209100	-2.68297600
C	-0.01502500	0.26596000	-3.20026700
H	0.14862200	0.32121700	-4.27335400
C	-0.13419300	1.50134200	-2.53026000
C	-0.36774800	-2.14820000	-3.67060700
H	-1.36299800	-2.61234300	-3.62768900
H	0.35423100	-2.93446300	-3.40925400
H	-0.17452200	-1.79887700	-4.68997600
C	-0.11776100	2.74212000	-3.38913500
H	-0.97670200	3.38717500	-3.15815500
H	-0.13036100	2.48128700	-4.45244600
H	0.78478000	3.33055500	-3.17470200
C	-0.96144200	-2.56031600	-1.01031300
C	-0.06443900	-3.61565300	-0.75166800
C	-0.58875400	-4.86466000	-0.40312400
H	0.09569600	-5.68979400	-0.19382600

C	-1.96200200	-5.07313700	-0.33169300
H	-2.35426300	-6.05790000	-0.07132200
C	-2.83420500	-4.01872000	-0.58171400
H	-3.91229200	-4.18260600	-0.51498000
C	-2.35614200	-2.74779700	-0.91458700
C	1.43915800	-3.41863400	-0.82209300
H	1.63727200	-2.42637700	-1.25009000
C	2.13087500	-4.46495000	-1.70127800
H	2.03562900	-5.47480100	-1.27420200
H	3.20451700	-4.23981900	-1.77914400
H	1.71392400	-4.49299300	-2.71879900
C	2.03135100	-3.43767000	0.58832600
H	1.49443100	-2.75095800	1.26255700
H	3.08876300	-3.13431400	0.57008000
H	1.95989700	-4.44821100	1.01516500
C	-3.34510000	-1.62453500	-1.18549100
H	-2.76990900	-0.70677900	-1.37932000
C	-4.19121200	-1.91449900	-2.43002800
H	-4.79862700	-2.82216900	-2.29074600
H	-3.56508100	-2.06147700	-3.32187600
H	-4.87677900	-1.07806600	-2.63182900
C	-4.23292500	-1.36369700	0.03389300
H	-4.93638000	-0.54333200	-0.17685500
H	-3.62216000	-1.08085700	0.90436600
H	-4.82812900	-2.25355000	0.29406800
C	-0.53526100	2.91639700	-0.65630000
C	0.52126400	3.82641600	-0.44630300
C	0.21308700	5.09796300	0.05051100
H	1.02002500	5.81633500	0.21096700
C	-1.09351600	5.45381900	0.35778900
H	-1.31394000	6.45001700	0.74561500
C	-2.11805400	4.52611600	0.19547300
H	-3.13655200	4.80118100	0.47021700
C	-1.86488300	3.24892000	-0.31264600
C	1.97909700	3.46183100	-0.68091500
H	2.01728600	2.47874400	-1.17203800
C	2.71835400	4.47812600	-1.55782000
H	2.21825100	4.64700900	-2.52222400
H	3.74038200	4.12639900	-1.76094900
H	2.80071100	5.45315400	-1.05429000
C	2.69619600	3.32021300	0.66683500
H	2.77423300	4.30093100	1.16417700
H	3.71043200	2.91832500	0.52463700
H	2.14806500	2.64023500	1.33164400
C	-3.00710800	2.26394600	-0.51755900
H	-2.59714100	1.25538500	-0.36909000
C	-3.56041400	2.32616500	-1.94559000
H	-4.40529200	1.62826900	-2.05587700
H	-2.80026700	2.04603400	-2.68982700
H	-3.92145700	3.33924700	-2.18361800
C	-4.11736400	2.43495000	0.51743200
H	-4.83869600	1.60921200	0.43111500

H	-4.67825000	3.37178300	0.37337300
H	-3.70059200	2.42018800	1.53559400
C	-0.84657700	0.67620500	6.49766200
C	-0.01708400	-0.35246400	6.99220600
H	0.11356900	-0.37682500	8.07077300
C	0.48650000	-1.45796000	6.29118600
C	-1.50540400	1.57250800	7.51589400
H	-2.59915400	1.51093400	7.42174700
H	-1.23698300	2.62025500	7.32777000
H	-1.21035000	1.29598500	8.53329500
C	1.03860300	-2.59083000	7.12113000
H	0.95391300	-2.36631600	8.18895600
H	2.09108800	-2.78034200	6.87115300
H	0.49338100	-3.51979600	6.90014700
C	-2.11731000	1.80455300	4.83156500
C	-1.79651500	3.16580300	4.68250800
C	-2.82238800	4.06057400	4.35557300
H	-2.58621100	5.11984600	4.23327200
C	-4.12581500	3.62148200	4.17328700
H	-4.91561600	4.33216600	3.92504200
C	-4.41545100	2.26236000	4.26774300
H	-5.43185200	1.92183600	4.07083600
C	-3.42356600	1.32934500	4.57680900
C	-0.37327300	3.68883000	4.78392900
H	0.26943900	2.85205700	5.09574900
C	-0.21883900	4.81157600	5.81562300
H	-0.82240500	5.68777900	5.53563200
H	0.83058000	5.13714600	5.86795400
H	-0.52836500	4.50294000	6.82416700
C	0.09075400	4.17931700	3.40509200
H	-0.43131300	5.11133300	3.13988800
H	-0.12915400	3.44799200	2.61264900
H	1.17219400	4.39014500	3.40200900
C	-3.73711100	-0.16107500	4.59427700
H	-2.86475300	-0.66637100	4.15014500
C	-3.93506100	-0.71415900	6.01039700
H	-4.73998900	-0.17051400	6.52999500
H	-3.02136300	-0.64666600	6.61744000
H	-4.21982900	-1.77648800	5.96488500
C	-4.93292600	-0.51608500	3.71153100
H	-5.00853800	-1.60829200	3.60600000
H	-4.82233400	-0.07949600	2.70818400
H	-5.88150300	-0.16530400	4.14660200
C	0.86604400	-2.84465200	4.39327600
C	2.23444000	-3.15175900	4.23586100
C	2.57549500	-4.42640000	3.77889300
H	3.62867800	-4.68849600	3.66025400
C	1.59575300	-5.36096300	3.45417300
H	1.88354900	-6.35104900	3.09645900
C	0.25265200	-5.01947800	3.55466600
H	-0.51407900	-5.74288900	3.26825900
C	-0.13814900	-3.76363500	4.03325300

C	3.32110500	-2.12149200	4.50108300
H	2.86875000	-1.28733100	5.05791800
C	4.48309600	-2.67142000	5.33269100
H	4.14386200	-3.11902600	6.27845500
H	5.19118100	-1.86466100	5.57121500
H	5.04113800	-3.44162700	4.77999700
C	3.83665400	-1.56169200	3.17011500
H	3.01935100	-1.20931100	2.52487400
H	4.37414300	-2.34321000	2.61001500
H	4.53189000	-0.72561700	3.34060800
C	-1.61694500	-3.43540600	4.15840800
H	-1.70684500	-2.42872800	4.59218500
C	-2.33146000	-4.39883700	5.11126300
H	-3.39278900	-4.12477900	5.20588600
H	-1.88252600	-4.38203700	6.11546700
H	-2.28704300	-5.43304100	4.73742700
C	-2.28501000	-3.42079000	2.78074700
H	-2.28413100	-4.42699700	2.33559300
H	-1.76482400	-2.74221700	2.08597500
H	-3.33151600	-3.08658900	2.86026200

Table S10

In=P-TS

Atomic Number	Coordinates (Angstroms) X	Y	Z
H	0.00000000	0.00000000	0.00000000
P	0.00000000	0.00000000	1.72357000
In	2.19608300	0.00000000	0.56833700
N	0.87908400	-0.23280100	-1.17557300
N	4.30200800	-0.30166600	0.20763800
C	5.07214400	0.65269100	-0.33615800
C	4.89325400	-1.56849800	0.51585600
H	0.71932000	-1.20865100	-1.44150200
H	0.87704300	0.32865200	-2.02914100
C	6.48437100	0.27124400	-0.72021800
C	4.71577400	1.98706700	-0.55569400
C	4.94576500	-2.58098600	-0.46392900
C	5.42315600	-1.77400000	1.80705800
Ga	-0.85454600	-2.10428200	1.47240600
H	6.48330900	-0.58457500	-1.40925200
H	7.04195300	-0.04835100	0.17266800
H	7.00510500	1.11401700	-1.18538400
C	3.60218300	2.73148400	-0.07717800
H	5.47823300	2.58798900	-1.04716200
C	4.33570400	-2.38451600	-1.84285800
C	5.55639800	-3.79464800	-0.13258600
C	5.29072200	-0.71283900	2.88759300
C	6.02936000	-3.00121900	2.09125300
N	-0.91019000	-3.38478200	2.96316900
N	-2.83367700	-2.01826000	1.27632100

Cl	-0.28619900	-3.40843500	-0.30695600
C	3.69990500	4.22778800	-0.27837700
N	2.55828000	2.19706400	0.51103200
C	3.00776800	-3.14118800	-1.94812900
C	5.28346400	-2.79719700	-2.97217900
H	4.11575000	-1.31318700	-1.96197700
C	6.10407400	-4.00410600	1.12873500
H	5.60417800	-4.59029100	-0.87888700
C	6.56865600	-0.53078300	3.70864500
C	4.10468400	-1.05317400	3.79927300
H	5.07949600	0.24766100	2.39303500
H	6.44781200	-3.17593400	3.08431300
C	0.13218400	-3.38133500	3.94452300
C	-1.90229500	-4.26909800	3.05526800
C	-3.41633200	-0.87342400	0.63543500
C	-3.58572600	-3.05493100	1.61702500
H	4.70236700	4.58235900	-0.00525500
H	3.55668500	4.44491000	-1.34807200
H	2.94639400	4.77806700	0.29593600
C	1.53809900	2.98021600	1.13474900
H	2.51043900	-2.92686900	-2.90629600
H	3.17568000	-4.22859900	-1.88711200
H	2.31289900	-2.87672500	-1.13831500
H	4.83941200	-2.55038200	-3.94740400
H	5.47120900	-3.88121500	-2.96294400
H	6.25566800	-2.28767900	-2.89957800
H	6.58205400	-4.95570900	1.36614900
H	6.45625400	0.31949900	4.39714200
H	6.78838400	-1.41813400	4.32087100
H	7.43836900	-0.33828900	3.06364900
H	3.95682200	-0.27646900	4.56422500
H	3.15770400	-1.16172400	3.24333000
H	4.28206100	-2.01021700	4.31321400
C	-0.04594700	-2.61479800	5.11454500
C	1.31663800	-4.11253800	3.72661500
C	-1.74215000	-5.43001100	4.00432800
C	-3.10922400	-4.16733400	2.34632000
C	-3.68611800	-0.88965500	-0.74858600
C	-3.66160700	0.28255900	1.41234300
C	-5.04474900	-3.06149400	1.23536900
C	0.43137400	3.44028800	0.39697600
C	1.60463400	3.15401300	2.53573800
C	-1.31514100	-1.81923600	5.37893800
C	0.97659100	-2.61041900	6.06881800
C	1.55242600	-4.87538500	2.43643200
C	2.30958800	-4.08037600	4.70978000
H	-1.62887800	-5.07023800	5.03665800
H	-0.82223500	-5.98293800	3.76341400
H	-2.60177400	-6.10590200	3.94935700
H	-3.82076600	-4.97509000	2.49838300
C	-3.31747600	-2.05689500	-1.65052200
C	-4.25685800	0.24949300	-1.32868400

C	-3.34072200	0.32349200	2.90045400
C	-4.22551600	1.39558700	0.78285100
H	-5.14197700	-3.14372800	0.14413900
H	-5.52429700	-2.11619200	1.52518300
H	-5.56998800	-3.89918100	1.70571400
C	0.25461300	3.14745700	-1.08370500
C	-0.55873900	4.16582800	1.06869800
C	2.74514900	2.54778800	3.34097700
C	0.58940300	3.87850800	3.16251800
C	-1.00978800	-0.33298500	5.58235100
C	-2.08539500	-2.38542100	6.57744000
H	-1.96411600	-1.90691200	4.49446400
C	2.14388200	-3.34078000	5.87636100
H	0.85097400	-2.02536000	6.98266000
C	2.09545100	-6.28722900	2.66884600
C	2.49956200	-4.07443400	1.53911500
H	0.59373500	-4.96201000	1.90514700
H	3.23409800	-4.64002900	4.55132000
C	-2.16450300	-1.64353900	-2.57347800
C	-4.49834600	-2.56208400	-2.48667500
H	-2.95102400	-2.88201100	-1.02321500
C	-4.53581900	1.37988500	-0.57375400
H	-4.47189500	0.24864400	-2.39936500
C	-3.04807600	1.73959400	3.39352400
C	-4.43955900	-0.33868500	3.73945000
H	-2.41964200	-0.25899100	3.05042700
H	-4.42090600	2.29748900	1.36216400
C	0.37293400	4.42258200	-1.92674900
C	-1.08721100	2.45263200	-1.35550500
H	1.06227600	2.46344800	-1.38410000
C	-0.47671800	4.40045600	2.43460400
H	-1.41909900	4.53674900	0.50734100
C	2.35968000	2.24693800	4.78808600
C	4.00387800	3.42133800	3.28953300
H	2.99515900	1.58100100	2.87020900
H	0.61884800	4.02675100	4.24187500
H	-0.32503100	-0.17818000	6.43065400
H	-0.55361500	0.10168300	4.67951200
H	-1.93795800	0.21518300	5.80311700
H	-3.02320600	-1.83010400	6.72817700
H	-1.49204100	-2.30326200	7.50125700
H	-2.33943500	-3.44541900	6.43378100
H	2.92978300	-3.32698700	6.63347400
H	1.45448400	-6.86248900	3.35360500
H	3.11041500	-6.26643500	3.09388400
H	2.15168000	-6.82940200	1.71393300
H	2.09309800	-3.06905500	1.34402000
H	3.48470900	-3.95685200	2.01666900
H	2.63434800	-4.57146200	0.56758900
H	-1.76979100	-2.51641900	-3.11441800
H	-2.50995900	-0.90122900	-3.31134800
H	-1.34469200	-1.19484700	-1.99776100

H	-4.19695400	-3.44556900	-3.06822900
H	-4.83535300	-1.79663500	-3.20190200
H	-5.36519700	-2.83962900	-1.87002900
H	-4.97859600	2.25940800	-1.04413100
H	-2.28031300	2.21814600	2.76746800
H	-3.95221100	2.36806000	3.39656500
H	-2.67086400	1.70883400	4.42452600
H	-4.19765500	-0.26144300	4.81125900
H	-5.41047400	0.15413300	3.57300600
H	-4.54769300	-1.40715800	3.50180900
H	0.28075200	4.18894800	-2.99798300
H	-0.42910700	5.13070000	-1.66719600
H	1.33236600	4.93275500	-1.76487300
H	-1.13261600	2.09229000	-2.39527300
H	-1.92568200	3.15232400	-1.21441700
H	-1.25019400	1.59779800	-0.68232100
H	-1.26092500	4.96364200	2.94291000
H	1.45440800	1.62448200	4.83439000
H	2.18359200	3.16877200	5.36249800
H	3.17660700	1.70876100	5.29001400
H	3.79865700	4.41141200	3.72534000
H	4.81995100	2.95633100	3.86393700
H	4.35955500	3.56918100	2.26071900

Table S11

Tl=P-TS

Atomic Number	X	Y	Z
H	-0.68770100	1.37699600	-1.22593100
P	-0.85998500	0.86405400	0.31260900
Tl	1.47199400	0.56370500	-0.52567700
N	0.38768100	1.45416800	-2.37539800
N	3.58989900	0.09265500	-0.99031900
C	4.52211800	1.03896600	-1.18920400
C	3.94074000	-1.28004600	-1.15618300
H	0.20524400	0.76462000	-3.11019500
H	0.66148400	2.31720600	-2.85236200
C	5.82823800	0.58403100	-1.80587600
C	4.44230600	2.38937500	-0.85285000
C	3.72460200	-1.92588900	-2.39032900
C	4.49879300	-1.97506900	-0.06170200
Ga	-1.98331400	-1.02147400	-0.39561000
H	5.64446300	0.02722300	-2.73543000
H	6.34707700	-0.10667400	-1.12459200
H	6.48158800	1.43768300	-2.01275800
C	3.49698600	3.09042900	-0.04273600
H	5.31441400	2.97838000	-1.13079500
C	3.12070900	-1.19028800	-3.57461900
C	4.06143600	-3.27875400	-2.50314500
C	4.67874100	-1.29806400	1.28691800

C	4.83175500	-3.32249700	-0.22310500
N	-2.30303300	-2.44352600	0.91660900
N	-3.89823200	-0.60641900	-0.65600200
Cl	-1.46658300	-2.16421300	-2.26723200
C	3.92464200	4.48132200	0.37338200
N	2.35472800	2.59246900	0.34005400
C	1.67037200	-1.63193600	-3.78366600
C	3.93814200	-1.37319000	-4.85602000
H	3.10932800	-0.11708800	-3.33099200
C	4.61376900	-3.97493300	-1.43344400
H	3.89078100	-3.79421900	-3.45073000
C	6.03847900	-1.58929700	1.92470000
C	3.53871600	-1.71212200	2.22310400
H	4.60835600	-0.21060200	1.13502300
H	5.26226400	-3.87368500	0.61553300
C	-1.32619000	-2.69593400	1.93209900
C	-3.40541100	-3.18420000	0.83547100
C	-4.27360500	0.68545500	-1.15786800
C	-4.80279200	-1.56765900	-0.51392000
H	4.96953400	4.46894000	0.71083900
H	3.86998400	5.14618200	-0.50262300
H	3.28762700	4.89158000	1.16562800
C	1.44776400	3.24943900	1.21131200
H	1.19420300	-1.05756000	-4.59350400
H	1.61959800	-2.70001200	-4.05003900
H	1.06270300	-1.50407600	-2.87648500
H	3.51970400	-0.75482800	-5.66342400
H	3.91926400	-2.41851100	-5.19905800
H	4.99010200	-1.08510900	-4.71352500
H	4.87422900	-5.02883600	-1.54203400
H	6.16007300	-0.99708500	2.84359900
H	6.13885300	-2.64924500	2.20265600
H	6.86445400	-1.33825300	1.24346700
H	3.62860500	-1.20786300	3.19686100
H	2.54689900	-1.47287500	1.80366300
H	3.56294800	-2.79952600	2.39801600
C	-1.47086900	-2.04694700	3.17719200
C	-0.22455800	-3.53437900	1.66888300
C	-3.46138100	-4.47386700	1.61367600
C	-4.53388600	-2.82013400	0.08051900
C	-4.41350500	0.89692800	-2.54467400
C	-4.43962600	1.74560900	-0.23782700
C	-6.21110200	-1.31819200	-0.99081900
C	0.58467800	4.25836800	0.73749800
C	1.32036400	2.74583300	2.52779700
C	-2.65840400	-1.14742300	3.48921800
C	-0.50015100	-2.27071300	4.15830300
C	-0.01349700	-4.17975300	0.31040000
C	0.71763300	-3.72926100	2.68330900
H	-3.33704300	-4.28362200	2.68864400
H	-2.62405500	-5.12065700	1.31195900
H	-4.40780500	-4.99781300	1.44492100

H	-5.35361100	-3.53417600	0.07392200
C	-4.08104200	-0.16872200	-3.57581000
C	-4.79156300	2.16901700	-2.98821300
C	-4.22575400	1.54306000	1.25638700
C	-4.81120700	2.99867800	-0.73299800
H	-6.21656100	-1.19623800	-2.08312200
H	-6.59988500	-0.38116000	-0.56772100
H	-6.87244400	-2.14656400	-0.71687400
C	0.61513200	4.76206100	-0.69693200
C	-0.36406200	4.78942600	1.61839300
C	2.22960100	1.62706400	3.01212500
C	0.34961300	3.29850600	3.36423300
C	-2.21847200	0.23584200	3.97525700
C	-3.59194100	-1.80537700	4.51245900
H	-3.23057400	-1.00605400	2.55979800
C	0.58679400	-3.10551700	3.91938800
H	-0.59765000	-1.77883100	5.12885600
C	0.29155600	-5.67668100	0.40813300
C	1.10370400	-3.45088500	-0.44396400
H	-0.93529400	-4.05772200	-0.27596800
H	1.57521500	-4.37812900	2.49357600
C	-2.77443600	0.20842500	-4.28703600
C	-5.20012500	-0.38190700	-4.59975400
H	-3.90384500	-1.11927600	-3.05351300
C	-5.00173000	3.21035300	-2.09505100
H	-4.90478800	2.34586100	-4.05968800
C	-3.80826900	2.83114500	1.96502300
C	-5.45041400	0.91423700	1.93051200
H	-3.39269300	0.83268700	1.37279500
H	-4.94574200	3.83064700	-0.04246000
C	0.99641000	6.24600100	-0.76298200
C	-0.73400400	4.53333700	-1.39074100
H	1.37616500	4.18381400	-1.24306700
C	-0.48458000	4.32124100	2.91994100
H	-1.03518500	5.57581500	1.26499400
C	1.62569700	0.81694600	4.15649500
C	3.61510400	2.16375100	3.39031100
H	2.37404200	0.93465600	2.16553200
H	0.23026600	2.91813900	4.37882900
H	-1.62144200	0.16668200	4.89823200
H	-1.62170000	0.75865300	3.21105900
H	-3.10253300	0.84951300	4.20499400
H	-4.46527800	-1.16434100	4.70481600
H	-3.07152600	-1.96595400	5.46941900
H	-3.95957800	-2.77985400	4.16130100
H	1.33550100	-3.26656900	4.69701500
H	-0.48132700	-6.21395800	0.97776600
H	1.25969500	-5.86082800	0.89785200
H	0.34451900	-6.11587600	-0.59846000
H	0.88326800	-2.37417000	-0.51657600
H	2.07188800	-3.56882200	0.06913000
H	1.20245100	-3.83373700	-1.47060100

H	-2.42336400	-0.62263000	-4.91631300
H	-2.92713300	1.09392900	-4.92478100
H	-1.98405900	0.44616900	-3.56149100
H	-4.93574400	-1.20446600	-5.28007400
H	-5.35731800	0.51647300	-5.21538400
H	-6.16040500	-0.62892000	-4.12382200
H	-5.29391200	4.19633900	-2.45940300
H	-2.95400200	3.30153200	1.45518300
H	-4.63564400	3.55550700	2.01749500
H	-3.49956900	2.61153300	2.99593100
H	-5.28103400	0.81746200	3.01441200
H	-6.34318900	1.54139200	1.77979100
H	-5.66208200	-0.09119300	1.53796000
H	1.07957800	6.57778300	-1.80875800
H	0.22554300	6.86411300	-0.27750300
H	1.95066400	6.44834900	-0.25773800
H	-0.67259000	4.81520400	-2.45319100
H	-1.52074900	5.15005000	-0.92894100
H	-1.04615100	3.48237800	-1.33030000
H	-1.23989200	4.74046900	3.58655400
H	0.62688500	0.44271900	3.88957500
H	1.54478100	1.41303300	5.07847100
H	2.26001200	-0.05080300	4.38594200
H	3.53465600	2.87003100	4.23108400
H	4.27991700	1.34035400	3.69564100
H	4.08976500	2.68800300	2.54862800

Table S12

B=P-Prod

Atomic Number	Coordinates (Angstroms) X	Y	Z
Ga	0.10467900	0.04817100	0.22495500
B	-0.07330400	0.00693800	3.94404300
Cl	2.36213600	0.14937300	0.22695600
N	1.22913100	0.67672700	3.73092600
H	1.71523200	0.54754800	2.85049800
H	1.88628400	0.71169200	4.50641300
P	-1.14928900	-0.15165100	2.20604300
H	-1.48206400	1.23215100	2.12348900
N	-0.23880900	-1.29347300	-1.20634800
N	-0.35476400	1.59636100	-0.94788400
N	-0.94056300	0.85950800	5.01393100
N	0.05796800	-1.43908000	4.68473600
C	-0.13882600	-0.98126100	-2.49636900
C	-0.03696000	0.33030900	-2.98349200
H	0.06246800	0.43154100	-4.06075000
C	-0.24772400	1.52232400	-2.27166400
C	-0.15384400	-2.08055600	-3.53231700
H	-0.97613900	-2.78671000	-3.35852900
H	0.77856800	-2.65896200	-3.46849800

H	-0.23974800	-1.65992400	-4.53945100
C	-0.40182800	2.77406600	-3.10200300
H	-1.29521400	3.33740900	-2.80024500
H	-0.47024600	2.52368700	-4.16563700
H	0.45992400	3.43669900	-2.94842100
C	-0.55235600	-2.65085700	-0.87201900
C	0.46057000	-3.63088400	-0.85300000
C	0.08977600	-4.95432500	-0.59728700
H	0.85924300	-5.72892600	-0.57718000
C	-1.24063900	-5.30237800	-0.38709000
H	-1.51162000	-6.34415700	-0.20588000
C	-2.22729300	-4.32133900	-0.41830900
H	-3.27267500	-4.60342500	-0.27522100
C	-1.90494600	-2.98064900	-0.65013100
C	1.91850200	-3.28426600	-1.11229800
H	1.96093400	-2.25896700	-1.50716100
C	2.58014000	-4.22475400	-2.12482800
H	2.68530100	-5.24062400	-1.71523200
H	3.59063800	-3.86474900	-2.36692100
H	2.00938900	-4.30440800	-3.06159100
C	2.70822500	-3.28352100	0.19578600
H	2.21145100	-2.66781700	0.95709400
H	3.71829100	-2.87643500	0.03626100
H	2.79553600	-4.30790200	0.59029300
C	-3.00532900	-1.93676600	-0.77955500
H	-2.53241800	-0.94644800	-0.71789200
C	-3.67803200	-2.03575100	-2.15440500
H	-4.10405600	-3.03969700	-2.30736100
H	-2.96541400	-1.83954800	-2.96865400
H	-4.49422700	-1.30301300	-2.23915400
C	-4.03478000	-2.01310400	0.34571400
H	-4.76437700	-1.19497900	0.24009700
H	-3.54252100	-1.90743800	1.32223700
H	-4.59819800	-2.95954700	0.32905400
C	-0.70478900	2.86034600	-0.37074300
C	0.26040900	3.88056200	-0.24528400
C	-0.15855900	5.11826100	0.25450400
H	0.56940400	5.92660000	0.35124400
C	-1.47220300	5.32912200	0.65424100
H	-1.77318800	6.29877100	1.05499600
C	-2.40283400	4.29801400	0.55753200
H	-3.42607500	4.46840800	0.89258800
C	-2.04579600	3.05483300	0.02838900
C	1.72433200	3.68315000	-0.61700000
H	1.81856200	2.73062000	-1.15895700
C	2.25964900	4.81167700	-1.50742800
H	1.61969900	5.00616100	-2.38016800
H	3.26693300	4.55924000	-1.86904200
H	2.34242500	5.75392300	-0.94515700
C	2.59925600	3.56911600	0.63589000
H	2.55821400	4.50336600	1.21785300
H	3.64490800	3.38223600	0.34881900

H	2.27594800	2.74168500	1.27929300
C	-3.08498900	1.95830800	-0.16927000
H	-2.59824400	0.99885400	0.06202200
C	-3.57510700	1.88387500	-1.62092300
H	-4.38320000	1.14091200	-1.70357600
H	-2.78086500	1.58343000	-2.31815200
H	-3.97619400	2.85722600	-1.94488100
C	-4.27188500	2.08309800	0.78026600
H	-4.90955200	1.19057400	0.69915700
H	-4.89918800	2.95583800	0.53941300
H	-3.93589900	2.17167500	1.82179600
C	-0.71801100	0.72350300	6.31890000
C	-0.04307700	-0.39040900	6.82035100
H	0.11071900	-0.47726100	7.89221000
C	0.20380200	-1.50089800	6.00588900
C	-1.22772300	1.74703300	7.29991900
H	-2.30592500	1.91680200	7.18283000
H	-0.73577500	2.71295300	7.12090700
H	-1.01906400	1.42369100	8.32540800
C	0.59388400	-2.78318800	6.69424900
H	0.35479100	-2.71845600	7.76158100
H	1.67266200	-2.95448600	6.58998300
H	0.08416400	-3.64991900	6.25655200
C	-1.87564500	1.88753400	4.64366200
C	-1.45294800	3.22181600	4.50058200
C	-2.41822500	4.19261100	4.20955700
H	-2.10436400	5.22952300	4.07295500
C	-3.76580700	3.86436400	4.11537700
H	-4.50520200	4.63897300	3.90420100
C	-4.16969300	2.54527300	4.30073500
H	-5.23007800	2.29126500	4.23829100
C	-3.23776800	1.53541100	4.55303100
C	-0.00160200	3.63030300	4.67163500
H	0.53676200	2.77529100	5.10158700
C	0.16730100	4.84013200	5.59662600
H	-0.25080400	5.75159200	5.14376500
H	1.23600300	5.02793100	5.77677900
H	-0.32494900	4.69987800	6.57045300
C	0.63342500	3.91172900	3.31336900
H	0.20380300	4.82107400	2.86721800
H	0.46438600	3.07257100	2.62470200
H	1.71931900	4.05368400	3.42005400
C	-3.70896400	0.10791100	4.78221700
H	-2.82076800	-0.53754100	4.75308100
C	-4.35074500	-0.04536000	6.16624300
H	-5.19900500	0.64730300	6.28312500
H	-3.63015900	0.15734800	6.97186700
H	-4.72700900	-1.06983600	6.30526400
C	-4.65691300	-0.36899000	3.68184600
H	-4.87964300	-1.43973700	3.81369100
H	-4.19698300	-0.23293600	2.69312400
H	-5.61595000	0.17225800	3.70310300

C	0.34184800	-2.65362900	3.96300600
C	1.68534600	-3.06473000	3.80905200
C	1.93428000	-4.30331100	3.20813100
H	2.96718100	-4.63382100	3.08264600
C	0.89852800	-5.10855900	2.76244200
H	1.11162000	-6.06553400	2.28256200
C	-0.41913900	-4.69872300	2.93794800
H	-1.22019700	-5.35137400	2.59545400
C	-0.73146600	-3.48562000	3.55869200
C	2.88426900	-2.26041600	4.30095300
H	2.50873800	-1.43619500	4.92334500
C	3.85378100	-3.10556400	5.14090900
H	3.35332900	-3.69228200	5.92349400
H	4.59895000	-2.45443300	5.62092700
H	4.40597300	-3.81464400	4.50666800
C	3.66349000	-1.64057400	3.13764700
H	3.02214100	-1.04869800	2.47559200
H	4.13141800	-2.42910200	2.52735600
H	4.46646300	-0.99095800	3.51909500
C	-2.19013600	-3.13927800	3.85354800
H	-2.34545400	-2.10246200	3.51815600
C	-2.54106500	-3.21975100	5.34668400
H	-3.62370500	-3.06296900	5.47268900
H	-2.03306000	-2.46221200	5.95727400
H	-2.30183500	-4.21741600	5.74865500
C	-3.17525800	-4.02119800	3.08659300
H	-3.18175600	-5.04946200	3.48184000
H	-2.93594000	-4.06206800	2.01735500
H	-4.19392900	-3.62083200	3.19409800

Table S13

Al=P-Prod

Atomic Number	X	Y	Z
Ga	-0.03858600	0.00729900	0.02509800
Al	0.11712800	0.03508300	3.81615000
Cl	2.22165200	0.02325600	0.09381600
N	1.66501900	0.88942700	3.48580300
H	2.11076400	0.82079500	2.57360700
H	2.34940000	1.17343100	4.18017300
P	-1.30717800	-0.26122100	1.95230300
H	-1.63528200	1.13267600	1.98399000
N	-0.44265600	-1.30066200	-1.40057000
N	-0.44248300	1.57701600	-1.11488900
N	-0.92421300	0.94467500	5.16501400
N	0.36435700	-1.56066100	4.88879200
C	-0.24782200	-0.99117200	-2.68002600
C	-0.07289800	0.32081500	-3.14804400
H	0.09575900	0.42870600	-4.21629500
C	-0.28932300	1.51438900	-2.43393200

C	-0.24431700	-2.09809600	-3.70436700
H	-1.19173600	-2.65377000	-3.68289300
H	0.54710500	-2.82182500	-3.46200800
H	-0.07829400	-1.70066200	-4.71084800
C	-0.38284400	2.78179900	-3.24662500
H	-1.28494100	3.34864700	-2.97776000
H	-0.39822500	2.55581600	-4.31776300
H	0.47602400	3.43088500	-3.03022100
C	-0.89485300	-2.61993500	-1.07218600
C	0.03880600	-3.64333200	-0.81979700
C	-0.44402200	-4.92101100	-0.51661600
H	0.26693700	-5.72248500	-0.30454600
C	-1.80912600	-5.18741000	-0.49802700
H	-2.16771400	-6.19367700	-0.27480800
C	-2.71750400	-4.16542400	-0.75668500
H	-3.78871300	-4.37761000	-0.73216800
C	-2.28343700	-2.86499200	-1.02939400
C	1.53337100	-3.38013500	-0.86202600
H	1.69365700	-2.37944300	-1.28674400
C	2.28340900	-4.39213700	-1.73334400
H	2.23241100	-5.40517600	-1.30647900
H	3.34579800	-4.11600700	-1.79959700
H	1.87792000	-4.44035700	-2.75492100
C	2.10784700	-3.37192600	0.55513500
H	1.53251900	-2.71331900	1.22620800
H	3.14876400	-3.01649600	0.54977200
H	2.08025600	-4.38492300	0.98035600
C	-3.30418800	-1.76899800	-1.29688800
H	-2.76212800	-0.81354200	-1.35899400
C	-4.01506900	-1.98591300	-2.63743900
H	-4.55812600	-2.94377400	-2.64209200
H	-3.30516700	-1.99495400	-3.47692800
H	-4.74411500	-1.18264500	-2.82173100
C	-4.31465400	-1.65043000	-0.15358600
H	-5.01695900	-0.82656200	-0.35426700
H	-3.79979500	-1.45031000	0.79785200
H	-4.90992100	-2.57039800	-0.04392600
C	-0.77543200	2.82975800	-0.50324900
C	0.21960100	3.80689900	-0.29369600
C	-0.16712700	5.02670900	0.27144900
H	0.58752200	5.79858500	0.43750300
C	-1.48193100	5.26113700	0.65202500
H	-1.75972500	6.21530300	1.10359300
C	-2.44263800	4.26911500	0.47733000
H	-3.46566400	4.45482200	0.80398800
C	-2.11595000	3.04379100	-0.11053900
C	1.68888500	3.57084800	-0.61358400
H	1.77715300	2.62432800	-1.16665700
C	2.29655100	4.69436300	-1.46187200
H	1.72022800	4.89695300	-2.37602800
H	3.32283400	4.43028300	-1.75552300
H	2.35110800	5.63476100	-0.89310900

C	2.49784200	3.41229700	0.67883500
H	2.51044400	4.36040500	1.24011400
H	3.53585900	3.13240500	0.44492000
H	2.07567700	2.63655500	1.33082500
C	-3.18466700	1.98410500	-0.34918500
H	-2.72560500	1.00316400	-0.15280400
C	-3.67042400	1.97175100	-1.80359900
H	-4.48007800	1.23462300	-1.92211200
H	-2.87074300	1.69687500	-2.50603500
H	-4.06459900	2.95909300	-2.09138100
C	-4.36688100	2.11349600	0.60799300
H	-5.02401200	1.23751300	0.51247300
H	-4.97530800	3.00478400	0.38810300
H	-4.02559500	2.17514800	1.65115400
C	-0.74087200	0.72100700	6.46619400
C	-0.06221300	-0.40030800	6.96349900
H	0.03348000	-0.47244200	8.04378300
C	0.35733300	-1.51920600	6.21922200
C	-1.28792600	1.70430800	7.47080100
H	-2.35792600	1.88957800	7.30762700
H	-0.77949800	2.67124500	7.34460100
H	-1.13067500	1.34667700	8.49385200
C	0.81289200	-2.72160900	7.00967100
H	0.60390000	-2.58426200	8.07560500
H	1.89228700	-2.87457500	6.87350900
H	0.32056700	-3.63659600	6.65317500
C	-1.87632300	1.94802600	4.78148300
C	-1.48493500	3.28976700	4.62419600
C	-2.45805200	4.22577300	4.25413000
H	-2.16679000	5.26917000	4.11408300
C	-3.78263100	3.85054400	4.06832300
H	-4.53002900	4.59579700	3.79027300
C	-4.15365200	2.51751900	4.22991900
H	-5.19368500	2.22690400	4.07192000
C	-3.21342700	1.54271100	4.57119600
C	-0.04809700	3.73992500	4.81906400
H	0.52304500	2.88457400	5.20747700
C	0.07339100	4.90165700	5.81069100
H	-0.41598600	5.80720100	5.42149600
H	1.13238300	5.14723700	5.97834500
H	-0.38368600	4.67215000	6.78423700
C	0.56669000	4.12326700	3.47267300
H	0.09928500	5.04054200	3.08220000
H	0.42477600	3.32263000	2.73373700
H	1.64798400	4.30038000	3.57760100
C	-3.63637600	0.09026400	4.74169800
H	-2.73884000	-0.52684900	4.59560200
C	-4.15155300	-0.18307500	6.16027000
H	-4.99169300	0.48618400	6.40426700
H	-3.36558500	-0.03975600	6.91491800
H	-4.50617600	-1.22129100	6.24445500
C	-4.66431400	-0.35007100	3.69911200

H	-4.81522000	-1.43855900	3.76130000
H	-4.31967200	-0.11074400	2.68285700
H	-5.64453300	0.12438600	3.86196800
C	0.74917500	-2.80104800	4.27194900
C	2.11242300	-3.15914000	4.18232500
C	2.42693500	-4.44280800	3.72858800
H	3.47467600	-4.74441100	3.66758300
C	1.43352500	-5.33483600	3.33749600
H	1.70200900	-6.33399700	2.98964400
C	0.10202600	-4.93631800	3.35347500
H	-0.67269100	-5.62365800	3.00954300
C	-0.26503800	-3.66998800	3.82236700
C	3.23599200	-2.19105000	4.52138000
H	2.79238500	-1.31645000	5.01911300
C	4.29473900	-2.79678500	5.44872100
H	3.86292800	-3.21332500	6.37013900
H	5.02841500	-2.02884200	5.73402400
H	4.84822200	-3.60472600	4.94734700
C	3.89777700	-1.69363800	3.23016800
H	3.16352100	-1.26155300	2.53709800
H	4.40533900	-2.52632800	2.71715500
H	4.65141000	-0.92367700	3.45666100
C	-1.73122200	-3.27926200	3.89753700
H	-1.77345400	-2.18450900	3.93605400
C	-2.38835900	-3.80567500	5.17715500
H	-3.45595700	-3.53699300	5.19630700
H	-1.91942200	-3.37865100	6.07663900
H	-2.31155600	-4.90284900	5.23657000
C	-2.49985300	-3.70046200	2.64772600
H	-2.61579700	-4.79353700	2.57658700
H	-1.98730000	-3.34806700	1.74052500
H	-3.50844200	-3.25967900	2.65642300

Table S14

Ga=P-Prod

Atomic Number	Coordinates (Angstroms) X	Y	Z
Ga	-0.05015000	0.02109100	0.00698300
Ga	0.11722800	0.03617400	3.75333800
Cl	2.20349200	0.01869500	0.13187100
N	1.71256900	0.97470300	3.50390300
H	2.21155400	0.80856600	2.63227500
H	2.38366600	1.04748600	4.26457900
P	-1.34889100	-0.25178000	1.91944600
H	-1.67546400	1.14126300	1.96140100
N	-0.44732900	-1.28965500	-1.41383900
N	-0.44101300	1.58953300	-1.13144400
N	-0.94564600	0.95784900	5.17836200
N	0.39315300	-1.59362700	4.89671300
C	-0.24731000	-0.98205000	-2.69324400

C	-0.06934900	0.32941800	-3.16202400
H	0.10376800	0.43572400	-4.22972300
C	-0.28377800	1.52501900	-2.45002700
C	-0.23775400	-2.09133100	-3.71473900
H	-1.18138700	-2.65327200	-3.69164600
H	0.55851400	-2.80899700	-3.46980400
H	-0.07380500	-1.69588900	-4.72232900
C	-0.36846900	2.79268300	-3.26262800
H	-1.27025000	3.36224700	-2.99841800
H	-0.37795300	2.56769700	-4.33402500
H	0.49108700	3.43871800	-3.03969900
C	-0.90404500	-2.60706000	-1.08235000
C	0.02506600	-3.63267000	-0.82239900
C	-0.46340500	-4.90840200	-0.51953800
H	0.24405600	-5.71162200	-0.30254500
C	-1.82927900	-5.17032300	-0.50610200
H	-2.19207000	-6.17505400	-0.28292000
C	-2.73331600	-4.14525300	-0.76795400
H	-3.80524100	-4.35366700	-0.74625000
C	-2.29383700	-2.84690500	-1.04168800
C	1.52087000	-3.37535100	-0.85358600
H	1.68820000	-2.37381700	-1.27368200
C	2.27267900	-4.38752400	-1.72327700
H	2.21409700	-5.40175400	-1.30031400
H	3.33670300	-4.11590000	-1.78106800
H	1.87422000	-4.43039800	-2.74782400
C	2.08527600	-3.37553900	0.56791600
H	1.51061000	-2.71379800	1.23638200
H	3.12904800	-3.02854900	0.57056500
H	2.04678100	-4.38893700	0.99136800
C	-3.30993800	-1.74790300	-1.31485300
H	-2.76613900	-0.79263600	-1.36305300
C	-4.00118200	-1.95388200	-2.66744500
H	-4.54098500	-2.91335900	-2.68862100
H	-3.27928800	-1.95266200	-3.49665800
H	-4.72985400	-1.15095900	-2.85456600
C	-4.33767300	-1.63590900	-0.18632200
H	-5.03295600	-0.80697900	-0.39019100
H	-3.83876000	-1.44756000	0.77599300
H	-4.93928400	-2.55358100	-0.09501300
C	-0.77278200	2.84284000	-0.51881600
C	0.22521800	3.81470700	-0.29999000
C	-0.16022200	5.03470400	0.26561000
H	0.59677800	5.80246700	0.43955800
C	-1.47625700	5.27349400	0.63894600
H	-1.75263600	6.22741600	1.09179100
C	-2.43962400	4.28528800	0.45804500
H	-3.46323300	4.47344300	0.78146900
C	-2.11424400	3.06022600	-0.13111300
C	1.69602400	3.57204500	-0.60685400
H	1.78515600	2.62317300	-1.15594600
C	2.31419400	4.68991100	-1.45507600

H	1.74845200	4.88839200	-2.37674400
H	3.34328400	4.42288100	-1.73591200
H	2.36380100	5.63322500	-0.89073500
C	2.49311400	3.41687000	0.69366400
H	2.50674500	4.36889700	1.24824800
H	3.53186400	3.13152300	0.46937400
H	2.06415100	2.64882400	1.35077400
C	-3.18551800	2.00415200	-0.37396000
H	-2.72849500	1.02123800	-0.18300600
C	-3.67065800	1.99746600	-1.82852300
H	-4.48140300	1.26208700	-1.95020300
H	-2.87065500	1.72387000	-2.53115800
H	-4.06276900	2.98651400	-2.11300900
C	-4.36739400	2.13150400	0.58422900
H	-5.02536700	1.25639600	0.48620100
H	-4.97505800	3.02378500	0.36667200
H	-4.02689300	2.19076400	1.62795800
C	-0.73222600	0.72626900	6.46828500
C	-0.02750900	-0.38809400	6.95381700
H	0.08287400	-0.45077900	8.03331300
C	0.38999200	-1.51795100	6.22334000
C	-1.27797600	1.69607400	7.48836600
H	-2.35120000	1.87237900	7.33607700
H	-0.77926100	2.66840300	7.36495100
H	-1.10834800	1.33215200	8.50719800
C	0.84919600	-2.70697400	7.03465200
H	0.65276200	-2.54793500	8.09989100
H	1.92595500	-2.87094300	6.88989300
H	0.34556900	-3.62510600	6.70204500
C	-1.90270900	1.95228100	4.79914400
C	-1.51908800	3.29618000	4.63593900
C	-2.49669300	4.22403200	4.25715000
H	-2.21151300	5.26890800	4.11504500
C	-3.81724300	3.83890500	4.06260600
H	-4.56790100	4.57798400	3.77688400
C	-4.17950300	2.50310000	4.22254600
H	-5.21543200	2.20434300	4.05370900
C	-3.23485700	1.53662900	4.57521800
C	-0.08415600	3.75431800	4.82623500
H	0.49677800	2.90108700	5.20446900
C	0.03362100	4.91170700	5.82345500
H	-0.46707600	5.81480700	5.44268500
H	1.09147700	5.16598000	5.98515500
H	-0.41460300	4.67268900	6.79881600
C	0.52139200	4.15004600	3.47903400
H	0.04687900	5.06720000	3.09657400
H	0.38321100	3.35253800	2.73586100
H	1.60231800	4.32930400	3.58041900
C	-3.64304300	0.07948200	4.74063800
H	-2.74315700	-0.52859500	4.56980300
C	-4.12430400	-0.21200400	6.16749100
H	-4.96138900	0.45101000	6.43752600

H	-3.32157600	-0.07325100	6.90504900
H	-4.47251100	-1.25262300	6.24880400
C	-4.69009300	-0.36045500	3.71719500
H	-4.82521100	-1.45157200	3.76744100
H	-4.37543500	-0.10193700	2.69565700
H	-5.67221500	0.09812200	3.91084800
C	0.76959800	-2.83799200	4.29333100
C	2.13062400	-3.20529300	4.20363000
C	2.43889400	-4.48854700	3.74492000
H	3.48476900	-4.79675300	3.68493300
C	1.44048100	-5.37252600	3.34726700
H	1.70336700	-6.37189100	2.99582700
C	0.11155500	-4.96483100	3.36078100
H	-0.66668900	-5.64506100	3.01053400
C	-0.24808500	-3.69790700	3.83359400
C	3.25525800	-2.23868400	4.54217500
H	2.81421700	-1.37464800	5.06088500
C	4.32702800	-2.85187400	5.44879900
H	3.90448900	-3.28484100	6.36700600
H	5.05931500	-2.08453700	5.73917000
H	4.87986600	-3.64888200	4.92959500
C	3.89736300	-1.71913100	3.24959700
H	3.15135700	-1.29044300	2.56628900
H	4.40689100	-2.54113300	2.72169000
H	4.64561300	-0.94343300	3.47434600
C	-1.71011900	-3.29109600	3.90024300
H	-1.74040500	-2.19554800	3.93851300
C	-2.37690000	-3.80681800	5.17912300
H	-3.44061500	-3.52312300	5.19715700
H	-1.90172200	-3.38553800	6.07804900
H	-2.31521600	-4.90487300	5.23915500
C	-2.47918000	-3.70628800	2.64856200
H	-2.61029200	-4.79794400	2.58165300
H	-1.95818700	-3.36626300	1.74125100
H	-3.48148900	-3.25120900	2.65081800

Table S15

In=P-Prod

Atomic Number	Coordinates (Angstroms) X	Y	Z
Ga	-0.11063700	0.19092800	-0.11888500
In	0.34833300	-0.00613000	3.62220800
Cl	2.13444400	0.36989800	0.01070700
N	2.18640900	0.94070400	3.53025400
H	2.62017300	0.91052700	2.60764400
H	2.88445100	0.66179500	4.21703700
P	-1.39744700	-0.11542200	1.79343900
H	-1.63153200	1.29015500	1.93980400
N	-0.43393900	-1.17931700	-1.48410100
N	-0.58284800	1.71132400	-1.28332900

N	-0.84144000	0.91312300	5.25098300
N	0.51537500	-1.76440000	4.98363700
C	-0.22408300	-0.89217400	-2.76960900
C	-0.12199900	0.41447900	-3.27030400
H	0.06373500	0.50305400	-4.33790400
C	-0.41703200	1.61973600	-2.59613800
C	-0.12371900	-2.03025200	-3.75376000
H	-1.06245600	-2.60034000	-3.78749200
H	0.65598700	-2.73358800	-3.42542900
H	0.11243800	-1.66238600	-4.75758600
C	-0.55434900	2.85711900	-3.44620400
H	-1.39125200	3.48098400	-3.10540200
H	-0.69619500	2.59109300	-4.49905100
H	0.35909500	3.46247300	-3.35766000
C	-0.87618200	-2.49598100	-1.13043300
C	0.05686200	-3.47262900	-0.72981100
C	-0.40931100	-4.75848500	-0.43843100
H	0.30084600	-5.52439800	-0.12151000
C	-1.75977900	-5.07453700	-0.55124700
H	-2.10523300	-6.08652700	-0.33328900
C	-2.67164100	-4.09167400	-0.92155200
H	-3.73387900	-4.33836000	-0.98576800
C	-2.25433600	-2.78646100	-1.20167700
C	1.52402300	-3.12586400	-0.56624100
H	1.72120400	-2.20094700	-1.12735600
C	2.46471800	-4.20779400	-1.09894900
H	2.40619400	-5.12774800	-0.49786900
H	3.50460000	-3.85397700	-1.05273400
H	2.23559500	-4.46805100	-2.14317100
C	1.81721200	-2.83988700	0.90996900
H	1.11658200	-2.09389500	1.32021700
H	2.83471400	-2.44412600	1.03401700
H	1.70633600	-3.75604300	1.50725400
C	-3.28738700	-1.73144900	-1.56941700
H	-2.76514900	-0.76909000	-1.68157100
C	-3.97058500	-2.04639900	-2.90438100
H	-4.51376600	-3.00262500	-2.85271300
H	-3.24439500	-2.11391300	-3.72699000
H	-4.69636700	-1.25973300	-3.15928800
C	-4.32232500	-1.56770500	-0.45215200
H	-5.05387400	-0.79110000	-0.72309300
H	-3.83647600	-1.27995900	0.49285100
H	-4.87926600	-2.50240000	-0.28357100
C	-0.95787300	2.94802100	-0.66167500
C	-0.00193500	3.96241500	-0.44870200
C	-0.42220600	5.13808300	0.18507700
H	0.30367900	5.93620500	0.35530200
C	-1.72913700	5.29610900	0.62399700
H	-2.03112300	6.21575000	1.12823600
C	-2.65190200	4.27070200	0.43502000
H	-3.66958700	4.39537800	0.80408700
C	-2.29241100	3.08696900	-0.21342300

C	1.46320400	3.82948400	-0.83869500
H	1.59630900	2.88252000	-1.38226100
C	1.92589000	4.98607700	-1.73453600
H	1.26938400	5.13850900	-2.60305200
H	2.94573800	4.79744300	-2.10020000
H	1.94891800	5.93220800	-1.17299000
C	2.35273700	3.76232900	0.40822200
H	2.28791300	4.70218500	0.97928000
H	3.40220100	3.60841100	0.11559400
H	2.06451700	2.93606100	1.07002300
C	-3.32380800	1.99281300	-0.45703300
H	-2.81894600	1.02309300	-0.32559400
C	-3.85319900	2.03032100	-1.89564400
H	-4.61543900	1.24891800	-2.04108900
H	-3.05493800	1.85601800	-2.63157100
H	-4.31758800	3.00494200	-2.11250600
C	-4.47494000	2.02969800	0.54620600
H	-5.09499400	1.12874000	0.43940600
H	-5.13001700	2.89958200	0.38269800
H	-4.09969000	2.06808800	1.57972100
C	-0.61023100	0.65966400	6.52935100
C	0.06488000	-0.48267600	7.00513800
H	0.16342400	-0.54324200	8.08629000
C	0.44417500	-1.64865300	6.30418100
C	-1.11427800	1.64080700	7.56365100
H	-2.19008500	1.82635500	7.44105200
H	-0.61099900	2.60823100	7.41555000
H	-0.91947200	1.28515000	8.58099100
C	0.77560900	-2.85267600	7.16056900
H	0.54037600	-2.65989800	8.21219100
H	1.84107600	-3.10783900	7.07428200
H	0.21355800	-3.73309600	6.81814500
C	-1.79475700	1.90305900	4.87266800
C	-1.39677200	3.23333300	4.64104800
C	-2.36340200	4.15793100	4.22950600
H	-2.06468300	5.19121800	4.03833800
C	-3.68894200	3.78031700	4.05311300
H	-4.43226500	4.51546200	3.73911400
C	-4.06365400	2.45418100	4.25967300
H	-5.10114200	2.15959100	4.09377200
C	-3.13042700	1.49208700	4.65346400
C	0.05542600	3.65919400	4.75527200
H	0.62861200	2.81696700	5.16905800
C	0.24607400	4.86864800	5.67439000
H	-0.25854700	5.75979900	5.27092500
H	1.31524900	5.10863300	5.76854700
H	-0.15527100	4.68690000	6.68226300
C	0.62101700	3.94388700	3.36179700
H	0.17571500	4.85841600	2.93841500
H	0.40282500	3.11764100	2.66717100
H	1.71350900	4.05793400	3.40244300
C	-3.54444200	0.03932800	4.84377100

H	-2.64941500	-0.57393200	4.65842800
C	-3.99384900	-0.23857900	6.28374700
H	-4.81918400	0.43319400	6.56798600
H	-3.17264400	-0.10137000	7.00059500
H	-4.34815300	-1.27589300	6.38126200
C	-4.61953900	-0.40416500	3.85081400
H	-4.77036000	-1.49180000	3.92463100
H	-4.32542500	-0.16852100	2.81709600
H	-5.59088400	0.07081200	4.05822600
C	0.81640900	-3.03524200	4.40569800
C	2.14044800	-3.52725200	4.38979900
C	2.36709600	-4.80588700	3.87003500
H	3.38254000	-5.20681000	3.86286500
C	1.32762800	-5.57001000	3.34874600
H	1.52680700	-6.56675000	2.95151000
C	0.03880200	-5.04620700	3.31124600
H	-0.77030300	-5.63195300	2.87147200
C	-0.23881800	-3.77875800	3.83252600
C	3.31937000	-2.69490500	4.87352400
H	2.92350900	-1.85512700	5.46398300
C	4.28276300	-3.48785600	5.76228700
H	3.76191000	-4.00058600	6.58425000
H	5.03601500	-2.81567300	6.19804600
H	4.82412300	-4.25114600	5.18375700
C	4.07200300	-2.09720400	3.67822900
H	3.42132200	-1.44020400	3.08449500
H	4.44948600	-2.89742900	3.02237900
H	4.93213100	-1.50277900	4.02333800
C	-1.65695600	-3.23403500	3.82332500
H	-1.58519500	-2.14026100	3.86170100
C	-2.42571300	-3.66981400	5.07456900
H	-3.45512400	-3.28004300	5.04661300
H	-1.94783200	-3.29014600	5.99057400
H	-2.47648700	-4.76801400	5.14044300
C	-2.41402300	-3.57231600	2.54166900
H	-2.65693400	-4.64425700	2.46875600
H	-1.82817000	-3.29154600	1.65286900
H	-3.36071400	-3.01186500	2.50741600

Table S16

Tl=Prod

Atomic Number	Coordinates (Angstroms) X	Y	Z
Ga	-0.15664200	0.25180900	-0.11465800
Tl	0.42595300	0.01041200	3.49975400
Cl	2.05862000	0.52945700	0.19052200
N	2.34188400	0.86050500	3.88663800
H	2.87527400	0.86582400	3.01470100
H	2.84909000	0.25822900	4.53701500
P	-1.44589800	-0.08273700	1.80811400

H	-1.66461700	1.32164500	1.97703800
N	-0.39536200	-1.13828800	-1.46407900
N	-0.62796100	1.75214400	-1.30461500
N	-0.85789000	0.88272500	5.25632800
N	0.51333700	-1.81807600	5.00947500
C	-0.17055000	-0.86032500	-2.75142800
C	-0.09942200	0.44031900	-3.26503500
H	0.09922500	0.52360700	-4.33133200
C	-0.44160400	1.64685400	-2.61197700
C	-0.01887900	-2.00788400	-3.71724600
H	-0.94284400	-2.60081000	-3.76478700
H	0.76953200	-2.68786000	-3.36203200
H	0.23120900	-1.64690900	-4.72019500
C	-0.59553100	2.85358200	-3.50394900
H	-1.19266600	3.64506100	-3.03635500
H	-1.05235600	2.56004500	-4.45686400
H	0.40349900	3.26030300	-3.72288000
C	-0.81487600	-2.46170500	-1.10495300
C	0.13143700	-3.41240800	-0.67585400
C	-0.31222900	-4.70501900	-0.37915700
H	0.40731500	-5.45027200	-0.03542600
C	-1.65235600	-5.05269900	-0.51806200
H	-1.97960600	-6.06962100	-0.29512200
C	-2.57799900	-4.09543100	-0.92025900
H	-3.63267700	-4.36726600	-1.00421900
C	-2.18444300	-2.78379400	-1.20367300
C	1.58820000	-3.03386700	-0.49040100
H	1.77134000	-2.10007300	-1.04201900
C	2.55834300	-4.09116200	-1.02040800
H	2.50820900	-5.01762600	-0.42885200
H	3.59040200	-3.71797600	-0.95515800
H	2.34979500	-4.34567500	-2.07029200
C	1.85760100	-2.75529300	0.99130600
H	1.13340000	-2.03098700	1.39848700
H	2.86413600	-2.33678100	1.12992700
H	1.76240300	-3.67825700	1.58148200
C	-3.23396800	-1.75509500	-1.59884100
H	-2.73133600	-0.78292800	-1.71778200
C	-3.89382600	-2.09994600	-2.93798500
H	-4.41749200	-3.06660600	-2.88154300
H	-3.15584400	-2.16201500	-3.75050100
H	-4.63275500	-1.33188800	-3.21116700
C	-4.28605800	-1.60024300	-0.49618300
H	-5.02549500	-0.83576400	-0.77969600
H	-3.81936800	-1.30311300	0.45561300
H	-4.83044200	-2.54248200	-0.32932700
C	-1.02318800	2.98284500	-0.68228400
C	-0.09191000	4.02412300	-0.49567600
C	-0.52707800	5.18389600	0.15810800
H	0.17894500	6.00388400	0.30669900
C	-1.82395900	5.30134300	0.63695800
H	-2.13721400	6.20872900	1.15604000

C	-2.72469200	4.25368300	0.46207700
H	-3.73633900	4.34963400	0.85495300
C	-2.35044200	3.08503100	-0.20365300
C	1.35300200	3.95460600	-0.96790200
H	1.50951200	2.99059700	-1.47412500
C	1.67436200	5.09000800	-1.94916100
H	0.94881000	5.14961400	-2.77214100
H	2.67758300	4.95080400	-2.37813800
H	1.66852900	6.06290800	-1.43459100
C	2.32546400	4.00194000	0.21542200
H	2.22666000	4.95015000	0.76705700
H	3.36210300	3.92311300	-0.14475500
H	2.14522700	3.17324400	0.91039200
C	-3.36366100	1.97589200	-0.45249100
H	-2.84445100	1.01060200	-0.33809500
C	-3.89274800	2.02796200	-1.89094100
H	-4.61801900	1.21729800	-2.06207600
H	-3.08483500	1.91633700	-2.62864000
H	-4.39963400	2.98692200	-2.07926700
C	-4.51449000	1.98199400	0.55150400
H	-5.12091800	1.07308100	0.43455300
H	-5.18231400	2.84348800	0.39646400
H	-4.14137900	2.01664700	1.58604500
C	-0.58327900	0.65304300	6.52652700
C	0.08907600	-0.49032000	7.00725300
H	0.20310000	-0.53417600	8.08806800
C	0.43958400	-1.67729200	6.32322300
C	-1.03873300	1.66351700	7.55858100
H	-2.11527900	1.86368000	7.46577700
H	-0.52535700	2.62011800	7.37617300
H	-0.81802900	1.32660800	8.57724100
C	0.72864000	-2.87816600	7.20436800
H	0.48869600	-2.66239500	8.25064400
H	1.78564600	-3.17080100	7.13565400
H	0.14074800	-3.74534800	6.87071800
C	-1.78099300	1.88529900	4.87262900
C	-1.34676000	3.19660800	4.59068100
C	-2.28908600	4.13755000	4.16210500
H	-1.96172400	5.15546900	3.93744500
C	-3.62827300	3.79571100	4.01037100
H	-4.35319000	4.54480800	3.68597200
C	-4.03889500	2.48637000	4.25406200
H	-5.08588300	2.21758900	4.10321200
C	-3.13000000	1.50903600	4.66712300
C	0.12242600	3.57093800	4.67168200
H	0.66526700	2.74008500	5.14490300
C	0.36708500	4.83356500	5.50158000
H	-0.09110500	5.71807700	5.03294200
H	1.44621300	5.02689500	5.58864400
H	-0.04842100	4.73975200	6.51569700
C	0.69969100	3.73529900	3.26239900
H	0.26606500	4.61485200	2.76007500

H	0.47484800	2.86238000	2.62590400
H	1.79301600	3.84272900	3.30222600
C	-3.57012500	0.06847500	4.88566000
H	-2.68805100	-0.56098100	4.68882000
C	-3.98920300	-0.18368300	6.33908800
H	-4.79836000	0.50330200	6.63360600
H	-3.14773500	-0.04688100	7.03198300
H	-4.35423100	-1.21487600	6.46064700
C	-4.67595900	-0.36652000	3.92349800
H	-4.85320600	-1.44834700	4.02092200
H	-4.39868800	-0.15810800	2.87883300
H	-5.63094100	0.13719300	4.13937700
C	0.78141400	-3.08985000	4.43840000
C	2.08712500	-3.63193400	4.43269600
C	2.28187200	-4.89933900	3.87324500
H	3.28338700	-5.33455300	3.87359900
C	1.23036900	-5.60826900	3.30099900
H	1.40500600	-6.59618300	2.87123300
C	-0.04055100	-5.04058900	3.26130000
H	-0.85998600	-5.58312500	2.78640200
C	-0.28598800	-3.78498700	3.82352300
C	3.28482900	-2.85706600	4.96347600
H	2.90515400	-2.01985800	5.56770700
C	4.19769200	-3.70724500	5.85235300
H	3.63728100	-4.21780400	6.64902800
H	4.96667900	-3.07621300	6.32116500
H	4.72098300	-4.47788300	5.26684600
C	4.08703600	-2.25600100	3.80294800
H	3.47088700	-1.56746400	3.20793000
H	4.45351900	-3.05076600	3.13431300
H	4.95682100	-1.69668200	4.18101300
C	-1.68310100	-3.18934700	3.81994100
H	-1.56613200	-2.09914200	3.86126200
C	-2.45844500	-3.59241400	5.07829200
H	-3.47293500	-3.16453500	5.05655200
H	-1.95946900	-3.22597700	5.98810700
H	-2.54894800	-4.68783200	5.14846500
C	-2.46518500	-3.50170600	2.54742900
H	-2.74814100	-4.56434400	2.47926600
H	-1.87845100	-3.24576800	1.65155900
H	-3.39146600	-2.90717600	2.52310000
